# Impaired mitophagosome–lysosome fusion mediates olanzapine‐induced aging

**DOI:** 10.1111/acel.14003

**Published:** 2023-10-13

**Authors:** Xi Chen, Zhizhen Wang, Peng Zheng, Anjila Dongol, Yuanyi Xie, Xing Ge, Mingxuan Zheng, Xuemei Dang, Zehra Boz Seyhan, Nathan Nagaratnam, Yinghua Yu, Xu‐Feng Huang

**Affiliations:** ^1^ School of Medical, Indigenous and Health Sciences University of Wollongong Wollongong New South Wales Australia; ^2^ Jiangsu Key Laboratory of Immunity and Metabolism, Jiangsu International Laboratory of Immunity and Metabolism, Department of Pathogen Biology and Immunology Xuzhou Medical University Xuzhou Jiangsu China

**Keywords:** accelerated aging, antipsychotics, cognition, health span, lifespan, mitophagy, mitophagy inducer, olanzapine

## Abstract

The lifespan of schizophrenia patients is significantly shorter than the general population. Olanzapine is one of the most commonly used antipsychotic drugs (APDs) for treating patients with psychosis, including schizophrenia and bipolar disorder. Despite their effectiveness in treating positive and negative symptoms, prolonged exposure to APDs may lead to accelerated aging and cognitive decline, among other side effects. Here we report that dysfunctional mitophagy is a fundamental mechanism underlying accelerated aging induced by olanzapine, using in vitro and in vivo (*Caenorhabditis elegans*) models. We showed that the aberrant mitophagy caused by olanzapine was via blocking mitophagosome–lysosome fusion. Furthermore, olanzapine can induce mitochondrial damage and hyperfragmentation of the mitochondrial network. The mitophagosome–lysosome fusion in olanzapine‐induced aging models can be restored by a mitophagy inducer, urolithin A, which alleviates defective mitophagy, mitochondrial damage, and fragmentation of the mitochondrial network. Moreover, the mitophagy inducer ameliorated behavioral changes induced by olanzapine, including shortened lifespan, and impaired health span, learning, and memory. These data indicate that olanzapine impairs mitophagy, leading to the shortened lifespan, impaired health span, and cognitive deficits. Furthermore, this study suggests the potential application of mitophagy inducers as therapeutic strategies to reverse APD‐induced adverse effects associated with accelerated aging.

## Graphical abstract

Olanzapine blocks the fusion between mitophagosomes and lysosomes, which inhibits the mitophagic flux (1), leading to shortened lifespan, and impaired health span, learning, and memory (2). By boosting mitophagy, the mitophagy inducer, urolithin A, ameliorates the lower mitophagic activity and adverse effects induced by olanzapine (3).

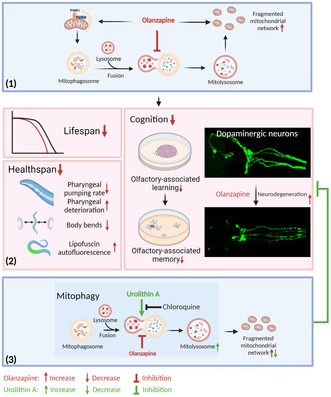

**Abbreviations**
AMPK: AMP‐activated protein kinase
APDs: antipsychotic drugs
ATP: adenosine triphosphate
BafA: bafilomycin A1
CQ: chloroquine
DMEM: Dulbecco's modified Eagle medium
LC3: light chain 3
MTR: Mitotracker
PFA: paraformaldehyde
ROS: reactive oxygen species
TFEB: transcription factor EB
VDAC: voltage‐dependent anion channel

## 1 INTRODUCTION

The lifespan of patients suffering from schizophrenia is about 14.5 years shorter compared with the general population (Hjorthøj et al., 2017). This increased mortality rate of patients is multifactorial, including accelerated aging, metabolic syndromes, and cardiovascular diseases (Constantinides et al., 2023; Hjorthøj et al., 2017; Nguyen et al., 2017). Therefore, identifying the underlying mechanism and developing interventions to reduce this mortality gap are urgently needed. In the clinical setting, all patients are under chronic, long‐term treatment schedules of antipsychotic drugs (APDs); however, it is unknown whether APDs contribute to increased mortality rates and lifespan shortening. Several follow‐up studies indicate that chronic administration of APDs contributes to abnormities in brain structure and cognitive performance, suggesting that administration of APDs may be involved in brain aging (Fusar‐Poli et al., 2013; Husa et al., 2014, 2017; Vita et al., 2015).

Olanzapine is one of the most efficacious APDs and is widely used for treating psychosis, including schizophrenia and bipolar disorders (Boz et al., 2020). However, olanzapine treatment has been found to cause alterations in brain structure, including the reduction of cortical thickness in both animal and human studies (Fusar‐Poli et al., 2013; Konopaske et al., 2007; Vernon et al., 2011; Voineskos et al., 2020). These findings suggest an association between olanzapine and brain aging (Fjell et al., 2015). Furthermore, olanzapine has been shown to impair learning and memory capacities in rodent models (Mutlu et al., 2011). However, there is no solid evidence demonstrating that the administration of olanzapine induces accelerated aging. Therefore, olanzapine in the context of accelerated aging and its potential underlying mechanisms need to be investigated.

Mitochondria are responsible for supplying the necessary energy (adenosine triphosphate, ATP) for cell survival and function, while progressive mitochondrial dysfunction is a hallmark characteristic of aging (Andrews et al., 2005; Miwa et al., 2022). Mitophagy is a selective type of autophagy that mediates the lysosomal clearance of damaged mitochondria to maintain mitochondrial homeostasis and quality (Ma et al., 2020). In this process, damaged mitochondria are engulfed in autophagosomes to form mitophagosomes, which are further fused with lysosomes to form mitolysosomes. Impaired mitophagy is related to the pathology of aging‐related, neurodegenerative, metabolic, and cardiovascular diseases (Ajoolabady et al., 2022; Fang et al., 2019; Shan et al., 2022; Xie et al., 2022). Previous studies have reported that olanzapine impairs mitochondrial function, affects mitochondrial cristae morphology, and triggers mitophagy initiation (Bar‐Yosef et al., 2020; Boz et al., 2020; Vucicevic et al., 2014). Furthermore, the accumulation of damaged mitochondria has been observed in olanzapine‐treated hypothalamic neurons, indicating a potential mitophagy impairment (Boz et al., 2020). However, the exact mechanism remains unclear. Therefore, olanzapine‐induced mitophagy impairments require further investigation.

Here we investigate whether defective mitophagy has a central role in olanzapine‐induced acceleration of aging. We explored the effect of olanzapine on the formation of mitophagosome and mitolysosome in cells and *Caenorhabditis elegans* (*C. elegans*). We examined the mitochondrial quality, quantity, and network morphology in response to olanzapine treatment. Additionally, we demonstrated learning and memory impairments, degenerated neurons, decreased neurite outgrowth, and reduced dendritic spine density using primary cortical neurons and *C. elegans* treated with olanzapine. Furthermore, a mitophagy inducer mitigated the effects of olanzapine on mitophagy and restored altered behaviors induced by olanzapine in *C. elegans*. We hypothesize that impaired mitophagy is a central mechanism underlying accelerated aging induced by the APD, olanzapine.

## 2 RESULTS

### 2.1 Olanzapine affects lifespan, health span, learning, and memory

**FIGURE 1:**
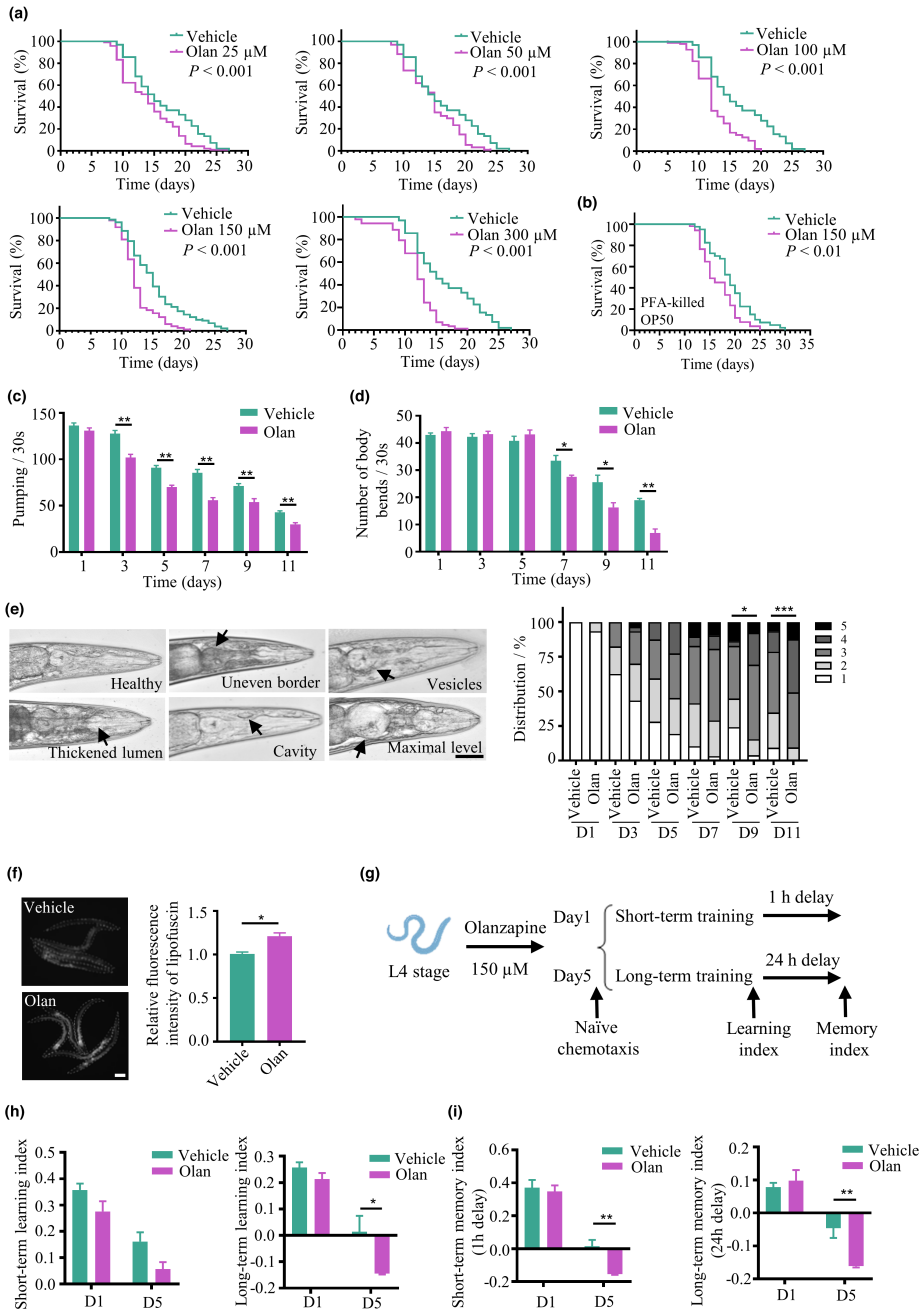
Olanzapine shortens lifespan, and impairs health span, learning, and memory in *C. elegans*. (a) Lifespan of worms treated with 25, 50, 100, 150, and 300 μM olanzapine (Olan) or vehicle. Data are representative of three independent experiments. ***p < 0.001, by log‐rank test. (b) Lifespan of worms fed with PFA‐treated OP50 and exposed to 150 μM olanzapine (Olan) or vehicle. Data are representative of three independent experiments. **p < 0.01, by log‐rank test. (c) Pharyngeal pumping at Days 1, 3, 5, 7, 9, and 11 of adulthood in worms treated with 150 μM olanzapine (Olan) or vehicle. *n* = 3 independent experiments. Values are mean ± SEM. **p < 0.01, by *t* test. (d) Body bends at Days 1, 3, 5, 7, 9, and 11 of adulthood in worms treated with 150 μM olanzapine (Olan) or vehicle. *n* = 3 independent experiments. Values are mean ± SEM. **p < 0.01, *p < 0.05, by *t* test. (e) Representative images of pharynx structure with healthy or deteriorated phenotypes and the classification of pharynx structure at Days 1, 3, 5, 7, 9, and 11 of adulthood in worms treated with 150 μM olanzapine (Olan) or vehicle. Scale bar = 40 μm. *n* = 26–40 per condition. ***p < 0.001, *p < 0.05, by Wilcoxon rank sum test. Arrows indicate deteriorated features in the pharynx. (f) Representative images of lipofuscin autofluorescence and quantification of fluorescence intensity at Day 9 of adulthood in worms treated with 150 μM olanzapine (Olan) or vehicle. Scale bar = 200 μm. *n* = 24–27 worms per condition. Values are mean ± SEM. *p < 0.05, by *t* test. (g) The schematic design of olfactory‐associated learning and memory assay conducted with 1‐day‐old or 5‐day‐old *C. elegans* treated with olanzapine or vehicle. (h, i) Quantification of short‐term learning, long‐term learning, short‐term memory, and long‐term memory index in *C. elegans* treated with olanzapine (Olan) or vehicle for 1 day or 5 days. *n* = 3–4 independent experiments based on more than 100 worms for each experiment. Values are mean ± SEM. **p < 0.01, *p < 0.05, by *t* test.

We found shortened longevity in *C. elegans* exposed to olanzapine over a range of doses (25, 50, 100, 150, and 300 μM), from the larval L4 stage until death, compared with the vehicle group (Figure 1a; Table S1). By feeding with paraformaldehyde (PFA)‐killed bacteria, olanzapine still decreased lifespan (Figure 1b; Table S1), suggesting the longevity regulated by olanzapine is independent of bacterial metabolism. Next, we examined whether olanzapine affects the health span of *C. elegans* treated with olanzapine, by assessing the pharyngeal pumping rate, body bending rate, pharyngeal deterioration, and lipofuscin autofluorescence intensity, which are commonly used parameters to assess the health span in *C. elegans* (Chen et al., 2019). The pharyngeal pumping rate started to reduce from day 3 of adulthood in nematodes treated with olanzapine from the L4 stage with a gradient reduction until late adulthood at Day 11, compared with the vehicle group (Figure 1c). Similarly, the frequency of body bending was significantly decreased from Day 7 to Day 11 following olanzapine administration (Figure 1d). Pharyngeal deterioration, which is another aging‐related phenotype, gradually reached a significant difference at Day 9 and 11 of adulthood in worms following olanzapine administration, compared with the vehicle group (Figure 1e). To further support that olanzapine accelerates aging, lipofuscin autofluorescence, a biological hallmark of aging, was measured in 9‐day‐old worms exposed to olanzapine from the L4 stage (Georgakopoulou et al., 2013). A significant increase in lipofuscin fluorescence intensity was observed in worms following olanzapine administration (Figure 1f). Given that chronic administration of APDs is related to poorer cognitive performance in schizophrenia patients, such as executive function, working memory, and verbal learning, here we investigated whether olanzapine induces learning and memory deficits in 1‐day‐old or 5‐day‐old *C. elegans* exposed to olanzapine from the L4 stage (Figure 1g; Husa et al., 2017). In the present study, olanzapine did not affect short‐term learning but dramatically impaired long‐term learning in 5‐day‐old worms (Figure 1h). Meanwhile, olanzapine treatment impaired both short‐ and long‐term memory in 5‐day‐old worms, but not in 1‐day‐old worms (Figure 1i).

### 2.2 Olanzapine impairs neurites

As aforementioned, olanzapine caused cognitive decline during aging in *C. elegans*, therefore, we investigated whether olanzapine leads to the degeneration of dopaminergic neurons, which are critical for adaptive learning and memory (Raj & Thekkuveettil, 2022). The age‐dependent damage of dopaminergic neurons (four CEP neurons) in *C. elegans* was assessed using the transgenic strain expressing *Pdat‐1::GFP* (Beilina et al., 2020). Olanzapine damaged neuronal morphology, including increased neurite blebbing, breaking, and absent axons, compared with the vehicle‐treated group (Figure S1a). Moreover, dendritic growth and synaptogenesis in cortical neurons are associated with cognitive function (Galakhova et al., 2022). Here, we investigated the effect of olanzapine on neurite outgrowth and spine density in primary cortical neurons. Indeed, olanzapine caused neurite lesions, including decreased total neurite length, reduced neurite branches in number and length, and lower synaptic spine density (Figure S1b,c).

### 2.3 Olanzapine damages mitochondria and impairs mitophagy

**FIGURE 2:**
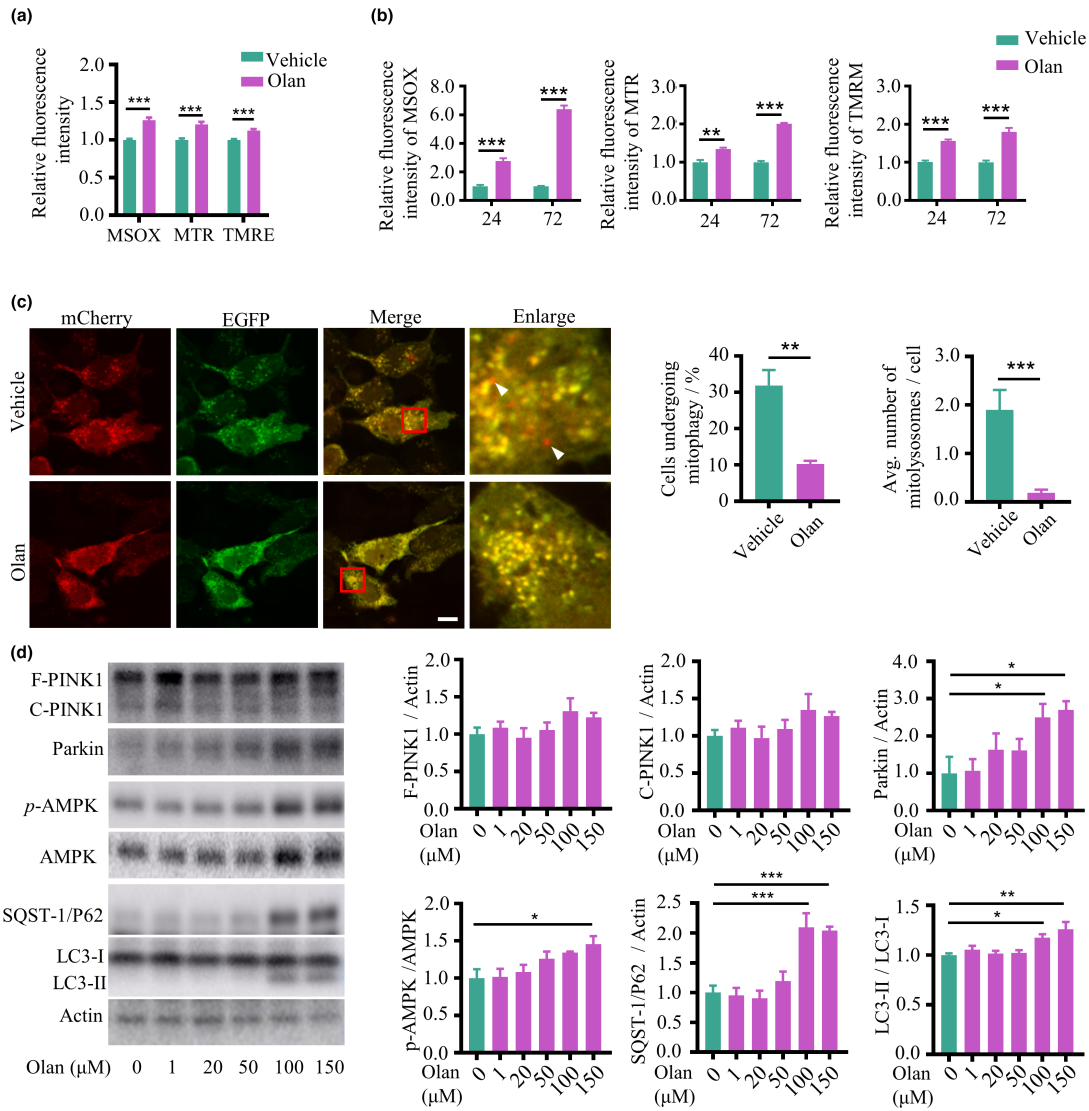
Olanzapine induces mitochondrial damage and impairs mitophagy. (a) Quantification of fluorescence intensity of MitoSOX (MSOX), Mitotracker green (MTR), and TMRE in *C. elegans* treated with olanzapine (Olan) or vehicle at Day 9 of adulthood. *n* = 20 for two independent experiments. Values are mean ± SEM. ***p < 0.001, by *t* test. (b) Quantification of fluorescence intensity of MSOX, MTR, and TMRM in HEK293T cells treated with olanzapine (Olan) or vehicle for 24 h. *n* = 6 biological replicates. Values are mean ± SEM. ***p < 0.001, **p < 0.01, by *t* test. (c) Representative images and quantification of HEK293T cells transfected with a mitophagy reporter (COX8‐EGFP‐mCherry) and treated with 150 μM olanzapine (Olan) or vehicle for 24 h. Scale bar = 10 μm. *n* = 102–122 cells from five representative images. Values are mean ± SEM. ***p < 0.001, **p < 0.01, by *t* test. Arrows indicate the mitolysosome (red‐only puncta). (d) Western blot plots and the quantification of full‐length or cleaved PTEN‐induced kinase 1 (PINK1), parkin RBR E3 ubiquitin‐protein ligase (Parkin), AMP‐activated protein kinase (AMPK), phosphorylated‐AMPK (*p*‐AMPK), microtubule‐associated protein light chain 3‐I/II (LC3‐I and LC3‐II), and sequestosome 1 (SQST‐1/P62) in HEK293T cells treated with olanzapine (Olan) ranging from 0 to 150 μM or vehicle for 24 h. *n* = 4 biological replicates. Values are mean ± SEM. ***p < 0.001, **p < 0.01, *p < 0.05, by one‐way ANOVA.

Mitochondrial quality and quantity control are governed by mitophagy and linked with age‐dependent pathologies, therefore, we first determined whether olanzapine affects mitochondrial‐related parameters (Ma et al., 2020). Olanzapine administration significantly increased mitochondrial reactive oxygen species (ROS), mitochondrial contents, and mitochondrial membrane potential in 9‐day‐old *C. elegans* (Figure 2a). The mitochondrial DNA to nuclear DNA (mtDNA/nDNA) between the groups treated with or without olanzapine in 5‐day‐old worms showed no difference, but decreased at Day 9 of adulthood (Figure S2a), suggesting long‐term exposure to olanzapine inhibits mitochondrial biogenesis.

Olanzapine increased mitochondrial ROS, contents, and membrane potential in HEK293T cells in a time‐dependent manner (Figure 2b). We noted that there were further increases in mitochondrial ROS and mitochondrial contents in cells over time, suggesting olanzapine disrupts mitochondrial homeostasis. Olanzapine increased mtDNA/nDNA ratio and mRNA expression of peroxisome proliferator‐activated receptor gamma coactivator (PGC‐1α), but not mitochondrial transcription factor A (TFAM; Figure S2b,c), which are mitochondrial biogenesis‐related genes (Ploumi et al., 2017). Together with the increased protein expression of mitochondrial biomarkers in cells exposed to olanzapine (Figure S2d), such as voltage‐dependent anion channel (VDAC) and cytochrome c oxidase subunit IV (COX IV), these results indicate the activation of mitochondrial biogenesis after acute treatment of olanzapine. The ATP level in HEK293T cells after treatment with olanzapine was shown a trend of decrease (*p* = 0.0950; Figure S2e), suggesting possible dysfunctional mitochondria triggered by olanzapine.

To demonstrate if olanzapine affects mitophagic flux, we transfected COX8‐EGFP‐mCherry, a mitophagy reporter, into HEK293T cells. The red‐only puncta representing mitolysosomes in HEK293T cells illustrates that mitochondria fused with lysosomal vesicles (Rojansky et al., 2016). We found that olanzapine decreased the percentage of cells undergoing mitophagy and inhibited the formation of mitolysosomes in each cell, compared with the vehicle group (Figure 2c). Furthermore, we measured the expression of mitophagy‐ and autophagy‐related proteins with PTEN‐induced kinase 1 (PINK1), parkin RBR E3 ubiquitin‐protein ligase (parkin), AMP‐activated protein kinase (AMPK), microtubule‐associated protein light chain 3 (LC3), and sequestosome 1 (SQST‐1/P62) using immunoblots (Seabright et al., 2020; Youle & Narendra, 2011). Our results showed that olanzapine increased the expression of parkin, phosphorylated‐AMPK, and lipidated LC3 (LC3‐II/LC3‐I) in a dose‐dependent manner, but not PINK1 (Figure 2d), which suggests the induction of mitophagy and autophagy. Olanzapine increased the ratio between full‐length PINK1 (F‐PINK1) and cleaved PINK1 (C‐PINK1) in the mitochondrial fraction of HEK293T cells (Figure S3a). Moreover, increased lipidated LC3 and SQST‐1/P62 conjugated to mitochondria were observed in olanzapine‐treated cells. Taken together, these results indicate that olanzapine stabilized F‐PINK1 on the mitochondrial membrane to initiate mitophagy. In addition, olanzapine treatment resulted in higher levels of SQST‐1/P62 (Figure 2d), an autophagic substrate degraded by autophagy (Mizushima et al., 2010). Taken together, olanzapine led to lower mitophagic flux and accumulated SQST‐1/P62 expression, indicating olanzapine triggers the mitophagic process, which does not reach completion. To further confirm this hypothesis, autophagy inhibitors—bafilomycin A1 (BafA) and chloroquine (CQ) were used. BafA blocks lysosomal vacuolar‐type H+‐ATPase (v‐ATPase), inhibiting the fusion between autophagosome and lysosome, whereas CQ serves as the lysosomotropic weak base, inducing lysosomal alkalization and inhibiting fusion (Redmann et al., 2017). The protein expression of LC3‐II/LC3‐I and SQST‐1/P62 were increased followed by olanzapine treatment; however, the addition of BafA and CQ did not show any difference compared with the olanzapine‐treated only group (Figure S3b), supporting that olanzapine blocks the degradation of autophagosomes. Furthermore, to investigate the role of mitophagy in olanzapine‐triggered lifespan shortening, the lifespan assay was performed in *C. elegans* in which mitophagy‐ and autophagy‐related genes, such as *pink‐1*, *dct‐1*, and *bec‐1*, were knockdown. The lifespan of *C. elegans* exposed to olanzapine was significantly shorter than the vehicle group, whereas no difference after the knockdown of *pink‐1*, *dct‐1*, and *bec‐1* (Figure S4; Table S1), suggesting the potential mechanism of impaired mitophagy underlying the shortened lifespan induced by olanzapine.

### 2.4 Olanzapine‐impaired mitophagosome–lysosome fusion is rescued by a mitophagy inducer

**FIGURE 3:**
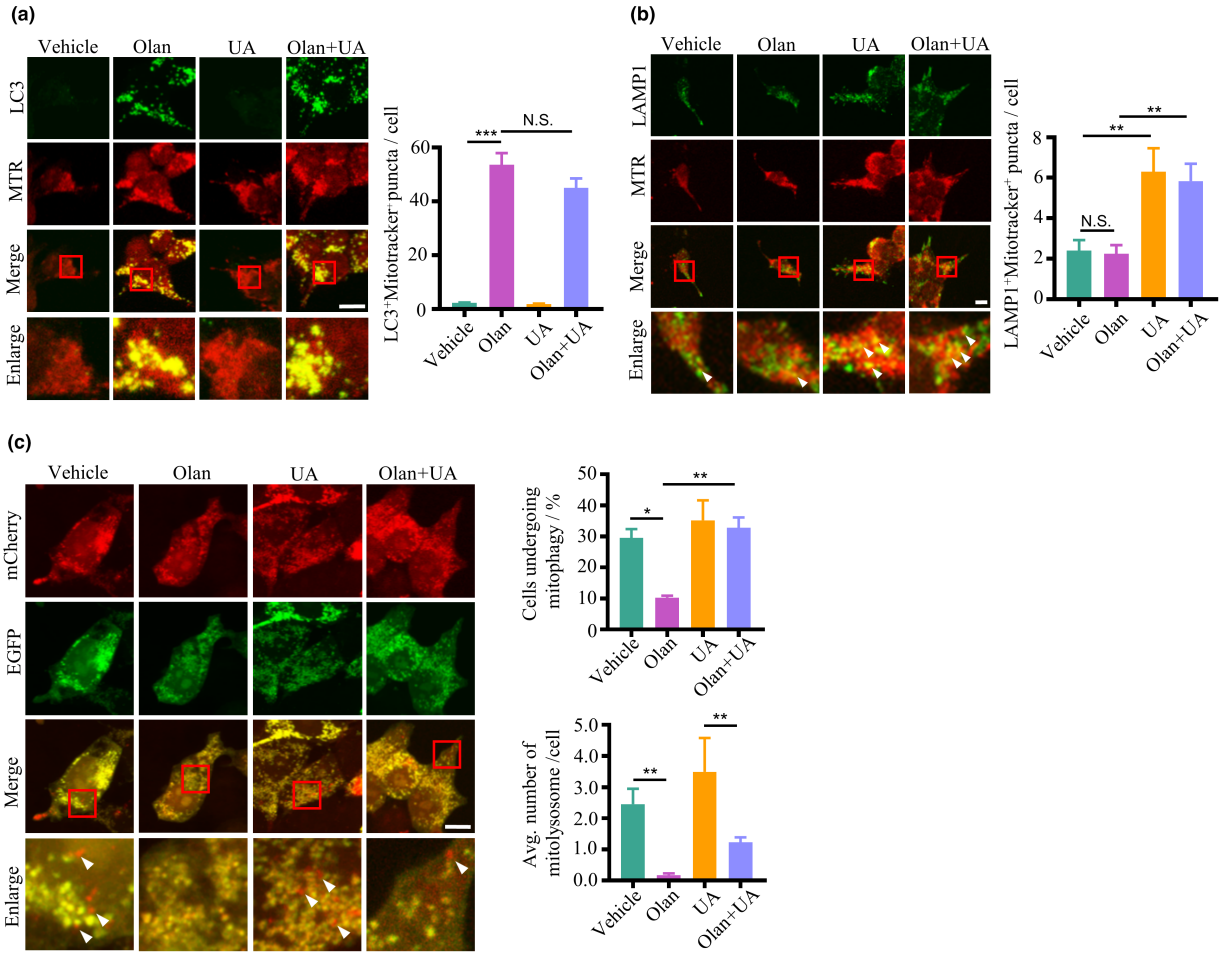
Olanzapine impairs mitophagosome–lysosome fusion, which is prevented by a mitophagy inducer. (a) Representative images and quantification of co‐localization between microtubule‐associated protein light chain 3 (LC3) and Mitotracker (MTR) in HEK293T cells treated with 150 μM olanzapine (Olan) in the absence or presence of 20 μM urolithin A (UA) for 24 h. Scale bar = 10 μm. *n* = 50 cells per group. Values are mean ± SEM. ***p < 0.001, N.S., not significant, by one‐way ANOVA. (b) Representative images and quantification of co‐localization between lysosomal‐associated membrane protein 1 (LAMP1) and Mitotracker (MTR) in HEK293T cells treated with 150 μM olanzapine (Olan) in the absence or presence of 20 μM urolithin A (UA) for 24 h. Scale bar = 10 μm. Values are mean ± SEM. *n* = 50 cells per group. **p < 0.01, N.S., not significant, by one‐way ANOVA. Arrows indicate LAMP1‐positive puncta co‐localized with MTR‐positive puncta. (c) Representative images and quantification of HEK293T cells transfected with a mitophagy reporter (COX8‐EGFP‐mCherry), treated with 150 μM olanzapine (Olan) in the absence or presence of 20 μM urolithin A (UA) for 24 h. Scale bar = 10 μm. *n* = 100–174 cells from six representative images. Values are mean ± SEM. **p < 0.01, *p < 0.05, by one‐way ANOVA. Arrows indicate the mitolysosomes (red‐only puncta).

The above experiment showed olanzapine caused incomplete mitophagy; however, it is not known whether this is due to the impaired fusion between mitophagosomes and lysosomes, thereby inhibiting the formation of mitolysosomes. First, we found that olanzapine initiated mitophagy, showing dramatically increased co‐localization between mitochondria (mitotracker‐positive puncta) and autophagosomes (LC3‐positive puncta) inside cells treated with olanzapine, which indicates an accumulation of mitophagosomes (Figure 3a). However, the co‐localization of mitochondria (mitotracker‐positive puncta) fused with lysosomes (lysosomal‐associated membrane protein 1 (LAMP1)‐positive puncta) inside cells had no differences between groups treated with or without olanzapine, suggesting unchanged mitolysosomes (Figure 3b). These results indicate that olanzapine blocks mitophagic flux. Urolithin A (UA) is a metabolite endogenously produced in the gut microbiome exposed to dietary polyphenols and has been identified as a mitophagy inducer (Ryu et al., 2016; Tomás‐Barberán et al., 2017). Here we hypothesized that UA could rescue the defective mitophagy caused by olanzapine. The activation of autophagy by UA has been confirmed by increased LC3‐II/LC3‐I and decreased SQST‐1/P62 (Figure S5a). Indeed, UA treatment increased the number of mitolysosomes in cells treated with olanzapine, but no influence was observed in the number of mitophagosomes when combined with olanzapine compared with olanzapine alone (Figure 3a,b), suggesting that UA ameliorates the impaired fusion between mitophagosomes and lysosomes induced by olanzapine. Additionally, UA counteracts the aberrant mitophagic flux induced by olanzapine (Figure 3c). These results demonstrate that UA promoting mitophagic activity in cells treated with olanzapine is dependent on mitophagosome–lysosome fusion.

### 2.5 Olanzapine‐induced mitochondrial fragmentation, lifespan shortening, and poor health span are ameliorated by a mitophagy inducer

**FIGURE 4:**
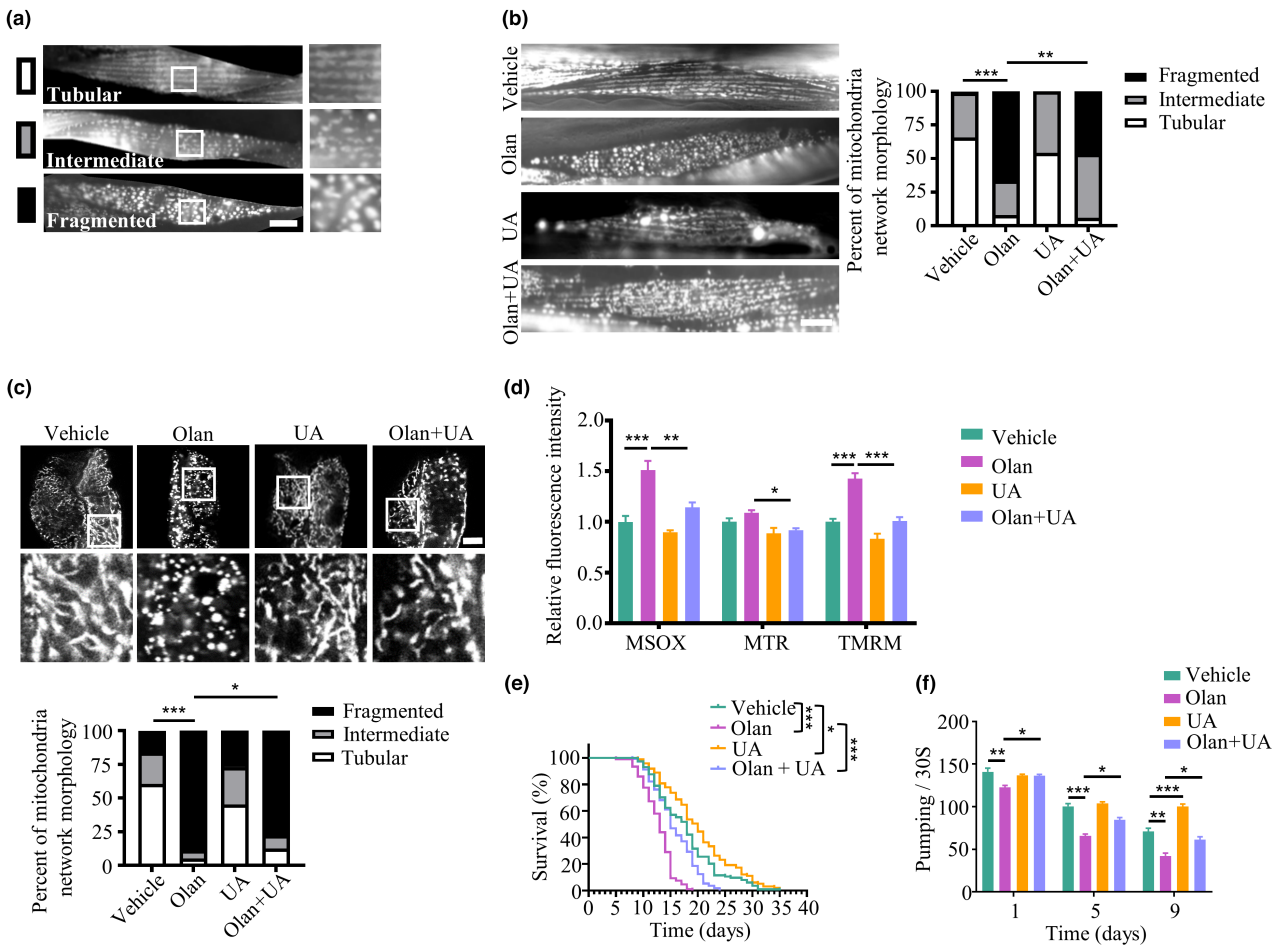
Olanzapine causes the hyperfragmented mitochondrial network, shortened lifespan, and impaired health span, which are mitigated by a mitophagy inducer. (a) Representative images of mitochondrial morphology show tubular (high degree of network connectivity throughout), intermediate (contained regions of both types), and fragmented (almost exclusively of isolated clusters of fluorescence with high circularity) phenotypes in body wall muscle cells of transgenic *C. elegans* expressing mitochondria‐targeted GFP. Scale bar = 10 μm. (b) Representative images and classification of mitochondrial morphology in body wall muscle cells of transgenic *C. elegans* treated with 150 μM olanzapine (Olan) in the absence or presence of 50 μM urolithin A (UA) at Day 9 of adulthood. Scale bar = 10 μm. *n* = 86–105 body wall muscle cells from 8 to 12 worms per group. ***p < 0.001, **p < 0.01, by chi‐squared test. (c) Representative images and classification of mitochondrial morphology in intestinal cells of transgenic *C. elegans* treated with 150 μM olanzapine (Olan) in the absence or presence of 50 μM urolithin A (UA) at Day 9 of adulthood. Scale bar = 10 μm. *n* = 81–152 intestine cells from 10 to 19 worms per group. ***p < 0.001, *p < 0.05, by chi‐squared test. (d) Mitochondrial parameters measured with MitoSOX (MSOX), Mitotracker (MTR), and TMRE in HEK293T cells treated with 150 μM olanzapine (Olan) in the absence or presence of 20 μM urolithin A (UA) for 24 h. *n* = 6 independent experiments. Values are mean ± SEM. ***p < 0.001, **p < 0.01, *p < 0.05, by one‐way ANOVA. (e) Lifespan of *C. elegans* treated with 150 μM olanzapine (Olan) in the absence or presence of 50 μM urolithin A (UA). Data are representative of three independent experiments. ***p < 0.001, *p < 0.05, by log‐rank test. (f) Pharyngeal pumping at Days 1, 5, and 9 of adulthood in worms treated with 150 μM olanzapine (Olan) in the absence or presence of 50 μM urolithin A (UA). *n* = 3 independent experiments with 20 worms per experiment. Values are mean ± SEM. ***p < 0.001, **p < 0.01, *p < 0.05, by one‐way ANOVA.

The integrity of the mitochondrial network is mediated by mitophagy and is critical for mitochondrial function (Sprenger & Langer, 2019). Given that olanzapine damaged mitochondria with defective mitophagy, we speculated that the mitochondrial network is hyperfragmented under the condition of olanzapine. The mitochondrial network was assessed using two transgenic worm strains with GFP‐labelled mitochondria in their body wall muscle cells and intestine cells. The mitochondrial morphology was assigned into tubular, intermediate, and fragmented, representing increasing levels of mitochondrial fragmentation (Figure 4a). Olanzapine administration significantly affected mitochondrial network morphology and induced mitochondrial hyperfragmentation in the muscle and intestine cells of *C. elegans* (Figure 4b,c; Figure S5b). Interestingly, the hyperfragmentation of mitochondrial network caused by olanzapine was partially ameliorated by UA treatments. Furthermore, UA prevented olanzapine‐induced changes in mitochondrial parameters in HEK293T cells, such as ROS levels, content, and membrane potential (Figure 4d). In *C. elegans*, progressive mitochondrial fragmentation is a common manifestation during aging, whereas inhibition of mitochondrial fragmentation is associated with lifespan extension (Lima et al., 2022). In addition to the previous finding that UA prevented mitochondrial hyperfragmentation induced by olanzapine, we found that UA rescued the shortened lifespan although the effects were relatively small (Figure 4e; Table S1). Moreover, UA ameliorated decreased pharyngeal pumping rates in nematodes exposed to olanzapine (Figure 4f).

### 2.6 CQ, but not BafA, blocks the effect of UA in preventing autophagy impairment induced by olanzapine

**FIGURE 5:**
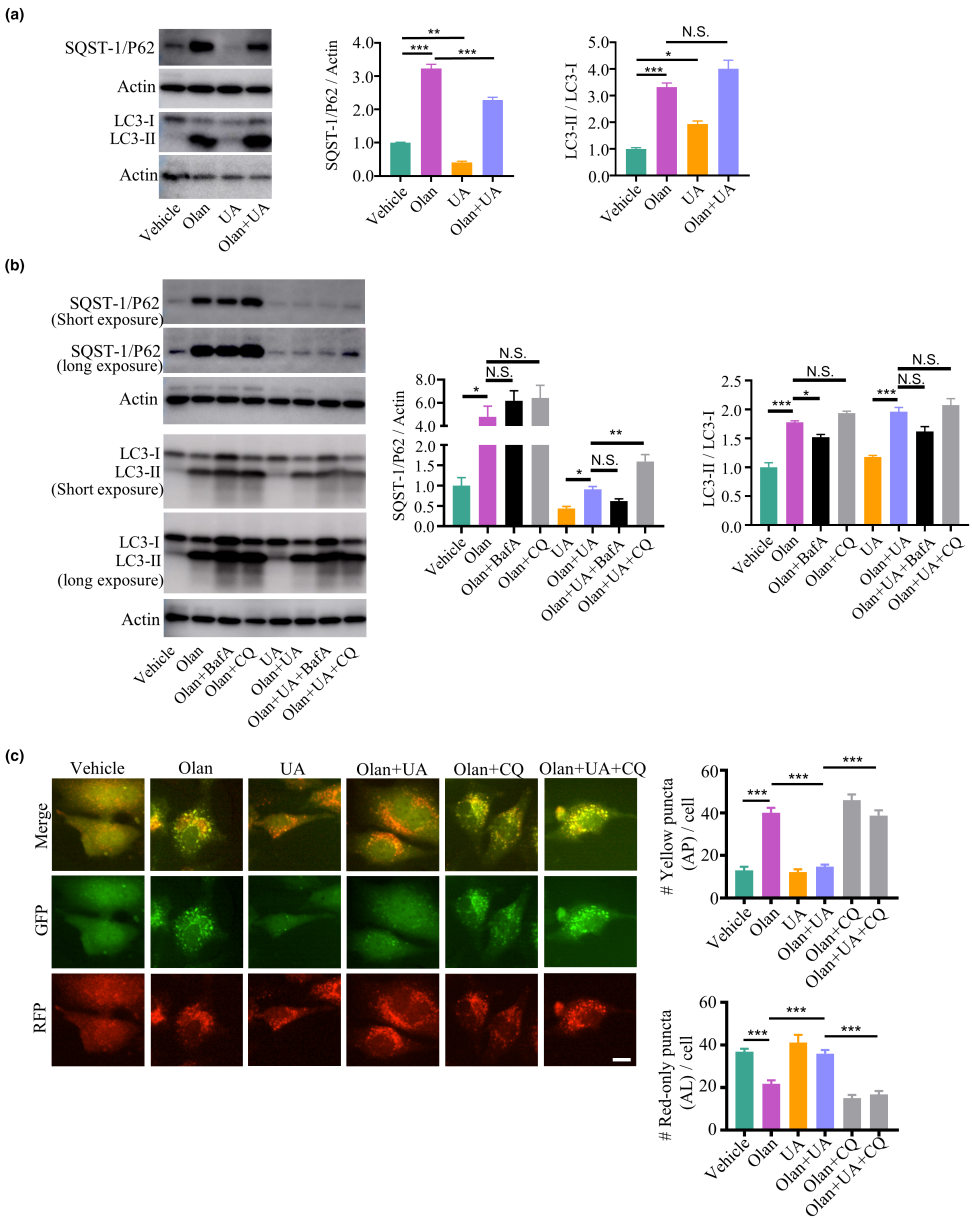
Defective autophagy induced by olanzapine is ameliorated by a mitophagy inducer. (a) Western blot plots and quantification of sequestosome 1 (SQST‐1/P62) and microtubule‐associated protein light chain 3‐I/II (LC3‐I and LC3‐II) in HEK293T cells treated with 150 μM olanzapine (Olan) in the absence or presence of 20 μM urolithin A (UA) for 24 h. *n* = 3 biological replicates. Values are mean ± SEM. ***p < 0.001, **p < 0.01, *p < 0.05, N.S., not significant, by one‐way ANOVA. (b) Western blot plots and quantification of SQST‐1/P62 and LC3 in HEK293T cells treated with 150 μM olanzapine (Olan) in the absence or presence of 20 μM urolithin A (UA) for 24 h with pre‐treatment of 200 nM bafilomycin A1 (BafA) or 50 μM chloroquine (CQ) for 6 h. *n* = 3 biological replicates. Values are mean ± SEM. ***p < 0.001, **p < 0.01, *p < 0.05, N.S., not significant, by one‐way ANOVA. (c) Representative images and quantification of autophagosome (AP, yellow‐puncta) and autolysosome (AL, red‐only puncta) in HeLa‐DiFluo cells treated with 150 μM olanzapine (Olan) in the absence or presence of 20 μM urolithin A (UA) for 24 h with pretreatment of 50 μM chloroquine (CQ) for 6 h. Scale bar = 20 μm. *n* = 20 cells per group. Values are mean ± SEM. ***p < 0.001, by one‐way ANOVA.

Fragmented mitochondria are degraded through autophagy machinery and as previously shown UA improved mitochondrial fragmentation, therefore, we hypothesized that UA can elevate autophagic activity (Sprenger & Langer, 2019). First, we found that UA facilitated SQST‐1/P62 degradation in cells treated with olanzapine (Figure 5a); however, UA did not affect the LC3‐II/LC3‐I ratio, indicating that UA enhances the fusion between autophagosomes and lysosomes. We found that CQ blocked SQST‐1/P62 degradation induced by UA, but did not affect LC3‐II/LC3‐I ratio, in olanzapine‐treated cells (Figure 5b). In contrast, BafA had no effects on either the SQST‐1/P62 or LC3‐II/LC3‐I ratio. Moreover, we quantified the number of autophagosomes and autolysosomes in HeLa cells expressing GFP (pH‐sensitive)‐RFP (pH‐insensitive)‐tagged LC3 (Liebl et al., 2022). Olanzapine increased the number of autophagosomes while decreasing the number of autolysosomes, which was counteracted by UA treatment. The administration of CQ blocked UA's effect on the number of autophagosomes and autolysosomes (Figure 5c). These results further consolidate that UA blocks olanzapine‐induced fusion impairment in the mitophagy process.

### 2.7 Restoration of autophagosome–lysosome fusion by a mitophagy inducer ameliorates learning and memory deficits in *C. elegans* exposed to olanzapine

**FIGURE 6:**
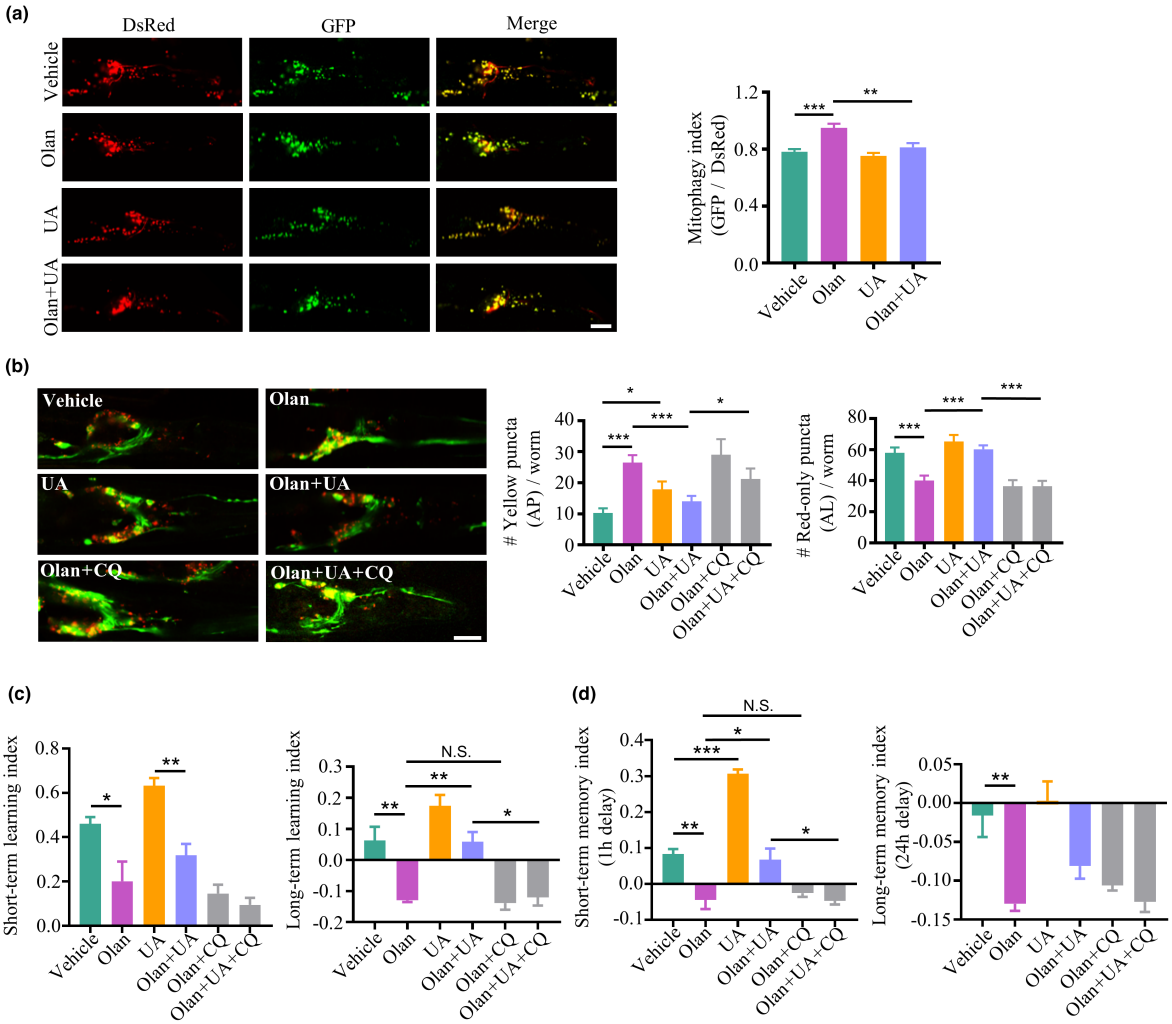
Restoration of autophagosome–lysosome fusion improves learning and memory in *C. elegans* exposed to olanzapine. (a) Representative images and quantification of neuronal mitophagy in *C. elegans* expressing mt‐Rosella reporter treated with 150 μM olanzapine (Olan), in the absence or presence of 50 μM urolithin A (UA) at Day 5 of adulthood. Scale bar = 10 μm. *n* = 20 worms per group. Values are mean ± SEM. ***p < 0.001, **p < 0.01, by one‐way ANOVA. (b) Representative images and quantification of the autophagosome (AP, yellow‐puncta) and autolysosome (AL, red‐only puncta) in transgenic *C. elegans* expressing *lgg‐1*‐RFP‐GFP treated with 150 μM olanzapine (Olan) in the absence or presence of 50 μM urolithin A (UA) with 5 mM chloroquine (CQ) at Day 5 of adulthood. Scale bar = 20 μm. *n* = 20 per condition. Values are mean ± SEM. ***p < 0.001, *p < 0.05, by one‐way ANOVA. (c, d) Short‐term learning, long‐term learning, short‐term memory, and long‐term memory index of *C. elegans* treated with 150 μM olanzapine (Olan) in the absence or presence of 50 μM urolithin A (UA) with 5 mM chloroquine (CQ) at Day 5 of adulthood. *n* = 3 independent experiments based on more than 100 worms for each experiment. Values are mean ± SEM. ***p < 0.001, **p < 0.01, *p < 0.05, N.S., not significant, by one‐way ANOVA.

Dysfunctional mitophagy drives neurodegeneration, which involves the manifestation of cognitive impairments; whereas enhancement of mitophagy restores cognitive deficits in animal models of neurodegenerative diseases (Jiao et al., 2022; Xie et al., 2022). As we found impaired learning and memory with degenerated neurons in *C. elegans* exposed to olanzapine, we investigated whether mitophagy was also affected by olanzapine in the neural system. We used the transgenic worm strain SJZ42, which expresses mitochondrial‐targeted Rosella in the pan‐neuronal system (Cummins et al., 2019). A mitophagy index is calculated by the ratio of fluorescence intensity between GFP and DsRed in SJZ42 worms. A higher GFP/DsRed ratio indicates lower mitophagic flux due to diminished GFP intensity, which is altered by the acidic environment. Notably, olanzapine inhibited the mitophagy flux, whereas UA alleviated lower mitophagic activity in nematodes exposed to olanzapine (Figure 6a). The general autophagy in the pan‐neuronal system of *C. elegans* was examined using the transgenic worm expressing RFP (pH‐insensitive) and GFP (pH‐sensitive) targeted with *lgg‐1* (homolog of LC3; Chang et al., 2017). Olanzapine increased autophagosomes while decreasing autolysosomes, which was reversed by UA treatment (Figure 6b). The administration of CQ blocked the protective effect of UA on olanzapine‐induced autophagy alterations in worms. These results suggest that UA improves mitophagic and autophagic flux in the neural system via enhancing fusion between autophagosomes and lysosomes in worms treated with olanzapine. Based on the above results, we examined whether UA and CQ affected short‐term and long‐term learning and memory in *C. elegans* exposed to olanzapine. Our data showed that UA did not improve short‐term learning, but did improve long‐term learning in worms exposed to olanzapine (Figure 6c). In addition, UA improved short‐term memory, but not long‐term memory (Figure 6d). Moreover, the enhancement of long‐term learning and short‐term memory induced by UA treatment was blocked by CQ administration in worms exposed to olanzapine. These results suggest that UA improves olanzapine‐induced learning and memory deficits through autophagosome–lysosome fusion in a time‐dependent manner.

## 3 DISCUSSION

In this study, we identified the role of defective mitophagy in accelerated aging induced by olanzapine. The aberrant mitophagy induced by olanzapine was via blocked fusion between mitophagosomes and lysosomes. Additionally, olanzapine treatments caused mitochondrial damage and hyperfragmented mitochondrial network. To confirm impaired mitophagosome and lysosome fusion in olanzapine‐induced aging models, we used a mitophagy inducer, UA, which restored defective mitophagy, mitochondrial fragmentation, and mitochondrial damage. In addition, UA prevented behavioral changes induced by olanzapine in *C. elegans*, including shortened lifespan, poor health span, and decline in learning and memory.

### 3.1 Olanzapine accelerates aging via defective mitophagy

Our data showed that olanzapine shortened longevity in *C. elegans* models from low to high concentrations, which is consistent with a previous single‐dose study (Weeks et al., 2010). Importantly, we, for the first time, showed that olanzapine impaired health span in *C. elegans*. Although previous studies have indicated that defective mitophagy plays a critical role in the etiology of aging and aging‐related diseases (Fang et al., 2019; Xie et al., 2022), no study has investigated the role of mitophagy in olanzapine‐induced accelerated aging. Our data showed that aberrant mitophagy is the partial underlying mechanism of accelerated aging induced by olanzapine. By boosting mitophagy, we showed that UA increased lifespan and improved health span in *C. elegans* treated with olanzapine, which supports that healthy mitophagy is beneficial for lifespan extension (Fang et al., 2019; Ryu et al., 2016). Based on the literature and our findings that UA cannot fully restore health span and lifespan in *C. elegans* exposed to olanzapine, olanzapine‐induced lifespan shortening involves multiple pathways. Previous studies have shown that olanzapine affects serotonin production through *tph‐1*, which may be associated with shortened lifespan and decreased reproduction period (Donohoe et al., 2008; Murakami & Murakami, 2007; Sze et al., 2000). Furthermore, olanzapine affects the insulin pathway via Akt/*daf‐2* contributing to the shortened lifespan in *C. elegans* (Weeks et al., 2010). Moreover, the knockdown of mitophagy genes, *pink‐1* and *dct‐1*, shortens the extended lifespan in *daf‐2* mutant worms, but not the wild‐type worms (Palikaras et al., 2015b). Taken together, the manifestation of lifespan shortening in *C. elegans* exposed to olanzapine may be due to an interaction between mitophagy and the insulin pathway.

Brain structure and cognitive performance can be affected in patients treated with APDs (Fusar‐Poli et al., 2013; Husa et al., 2017; Vita et al., 2015; Voineskos et al., 2020). Furthermore, rodent studies showed that olanzapine decreased cortical thickness and grey matter volume, which is associated with declined cognition (Milstein et al., 2013). Similarly, our study showed that olanzapine impaired learning and memory in *C. elegans*. Neurodegeneration is the hallmark of brain aging associated with cognitive decline, and can be investigated using *C. elegans* models (Cooper et al., 2015; Peng et al., 2011). In addition, dopaminergic neurons in *C. elegans* are responsible for adaptive learning and memory (Raj & Thekkuveettil, 2022). We found that olanzapine caused the degeneration of dopaminergic neurons in *C. elegans*. Furthermore, we showed that olanzapine reduced neurite outgrowth, branching arborization, and dendritic spine density in primary cortical neurons, which are all associated with cognitive function (Galakhova et al., 2022). These data suggest that neuronal lesions caused by olanzapine may contribute to cognitive decline. Since mitophagy integrity is important in maintaining neural function, we examined mitophagic flux. We found that *C. elegans* treated with olanzapine had a low mitophagic flux, supporting the idea that neural lesion is associated with mitophagy deficits (Zeng et al., 2022). Together, these results highlight that defective mitophagy may contribute to neuronal deficits, leading to cognitive decline. In addition, we demonstrated that mitophagy inducer, UA, improved learning and memory in *C. elegans* exposed to olanzapine.

### 3.2 Olanzapine impairs mitophagy via blocking mitophagosome–lysosome fusion

Here, we reported that olanzapine triggered mitophagy initiation, evidenced by increased parkin protein expression and mitophagosomes, which is consistent with previous literature (Vucicevic et al., 2014; Xiong et al., 2020). In regards to the completion of mitophagy, our results show that olanzapine blocked the mitophagosome and lysosome fusion, suggesting that olanzapine causes incomplete mitophagy.

Our study showed that olanzapine elevated phosphorylated AMPK and lipidated LC3, which are essential for the maturation of autophagosomes (Fritzen et al., 2016; Jang et al., 2018). This indicates olanzapine potently initiated autophagy, which is consistent with previous studies (Vucicevic et al., 2014; Xiong et al., 2020). The SQST‐1/P62 is an autophagic adaptor that recognizes autophagic cargos, mediates its engulfment into autophagosomes, and is degraded inside acidic lysosomal vesicles (Lippai & Lőw, 2014). Although previous studies have reported that olanzapine affects SQST‐1/P62 expression, the outcomes are inconsistent (Boz et al., 2020; Vucicevic et al., 2014; Xiong et al., 2020). Our results showed that olanzapine induced higher protein expression of SQST‐1/P62. Taken together, the accumulation of SQST‐1/P62 may be caused by autophagy initiation or autophagy incompletion, therefore, the use of SQST‐1/P62 as a predictor for autophagy completion is controversial, and whether autophagy is completed requires further investigation.

The autophagosome and lysosome fusion is the last step of autophagy required for completing the clearance of damaged organelles. However, we showed that olanzapine suppressed the autophagic flux by decreasing the number of autolysosomes, while increasing the number of autophagosomes. Previous studies demonstrated that CQ and BafA are autophagy inhibitors blocking the fusion between autophagosome and lysosome through lysosomal alkalization (Fedele & Proud, 2020; Redmann et al., 2017). We showed that CQ and BafA did not affect expressions of LC3‐II and SQST‐1/P62 in cells treated with olanzapine, suggesting that olanzapine may block the fusion between autophagosomes and lysosomes by affecting the lysosomal pH. APDs are weak base amphiphiles, which can penetrate membranes and be protonated inside lysosomes (Nadanaciva et al., 2011). The ionized form of APDs loses their permeability and becomes trapped within the lumina of lysosomes, which can affect the lysosomal function and morphology (Vantaggiato et al., 2019). Previous studies report that APDs, such as haloperidol and perphenazine, can cause lysosomal alkalization, lysosomal membrane damage, and dysfunctional lysosomal proteases, ultimately inhibiting autophagic flux (Canfrán‐Duque et al., 2016; Tao et al., 2022; Varalda et al., 2020). Therefore, the inhibited fusion between autophagosome and lysosome induced by olanzapine may be due to lysosomal alkalization and reduced lysosomal protease activity, which needs to be further investigated.

Recent studies show that UA initiates mitophagy via stabilizing PINK1 and activates autophagy, thereby prolonging lifespan in normal *C. elegans* and improving muscle function in *C. elegans* and rodents with muscular dystrophy (D'Amico et al., 2021; Luan et al., 2021; Ryu et al., 2016). Our study showed that olanzapine suppressed autophagic flux in cells and *C. elegans*, which was reversed by UA. To further understand UA's effect, we tested two different autophagosome and lysosome fusion inhibitors, CQ and BafA, under the condition of UA. We found that CQ, but not BafA, counteracted the effect of UA on autophagic flux in olanzapine‐treated models. Therefore, the effect of UA on the restoration of impaired autophagy could be associated with the mode of action of CQ. CQ was suggested to interfere with the proper recruitment of synaptosome‐associated protein 29 (SNAP29) onto autophagosomes, resulting in blocked fusion between autophagosomes and lysosomes, rather than altering lysosomal acidity (Mauthe et al., 2018). This implies the action mode of UA on the improvement of autophagosome–lysosome fusion may be via enhancing the translocation of SNAP29 onto autophagosomes, which requires further investigation. Furthermore, other possible factors regulating autophagy should not be excluded. For example, studies show that UA increases the nuclear translocation of transcription factor EB (TFEB), a crucial transcription factor for the regulation of autophagy (Settembre et al., 2011; Wang et al., 2019). The TFEB targets the promoter site of autophagy‐associated genes, regulating the formation of autophagosomes, autophagosome–lysosome fusion, lysosomal enzymes, and lysosomal degradation (Li et al., 2022; Settembre et al., 2012; Zhang et al., 2020; Zhao et al., 2020). Therefore, whether UA improves autophagosome–lysosome fusion in olanzapine‐treated models through TFEB activation needs further investigation.

### 3.3 Mitochondrial dysfunction induced by olanzapine associated with accelerated aging

Persistent oxidative stress inside mitochondria is implicated in aging and aging‐related diseases (Chistiakov et al., 2014). Previous studies reported olanzapine caused mitochondrial oxidative stress with abnormal morphology of mitochondrial matrix and cristae (Boz et al., 2020; Eftekhari et al., 2016; Salimi et al., 2018; Vucicevic et al., 2014). These are consistent with our results that olanzapine increased mitochondrial ROS levels and shortened lifespan in *C. elegans*. Additionally, it has been shown that olanzapine impaired mitochondrial function, such as oxygen consumption and ATP synthesis, in peripheral blood mononuclear cells derived from schizophrenia patients (Scaini et al., 2018). Mitochondrial dysfunction is considered to be triggered by accumulated mitochondrial oxidative damage, thereby leading to accelerated aging (Amorim et al., 2022; Kubben & Misteli, 2017; Miwa et al., 2022). These data suggest that accelerated aging induced by olanzapine may attribute to cumulative mitochondrial ROS production and mitochondrial dysfunction.

Mitochondrial dysfunction triggers compensatory mitochondrial biogenesis and mitophagy for the degradation of damaged organelles (Palikaras & Tavernarakis, 2014). A previous study reported that olanzapine downregulated PGC‐1α, a mitochondrial biogenesis biomarker, suggesting that olanzapine inhibits mitochondrial biogenesis (Liu et al., 2023). Decreased mtDNA content in *C. elegans* was found after long‐term exposure to olanzapine, supporting the suppressed mitochondrial biogenesis triggered by olanzapine. Additionally, olanzapine‐induced impaired mitophagy has been confirmed in our study. Taken together, olanzapine disrupts mitochondrial homeostasis, which is maintained by mitochondrial biogenesis and mitophagy (Palikaras et al., 2015a). Furthermore, studies show that mitochondrial biogenesis is upregulated following UA treatment, evidenced by the increased mRNA and protein levels of PGC‐1α (Kshirsagar et al., 2021b; Wu et al., 2022). As both mitochondrial biogenesis and mitophagy are enhanced by UA treatments, UA may restore mitochondrial homeostasis to preserve mitochondrial function in olanzapine‐treated models.

### 3.4 Olanzapine affects mitochondrial dynamics associated with aging

The mitochondrial network is dynamic and regulated by mitochondrial fusion and fission (Sharma et al., 2019). The hyperfragmentation of the mitochondrial network emerges with aging and is negatively regulated by fusion‐related proteins and positively regulated by fission‐related proteins (Chan, 2012; Jiang et al., 2015; Sharma et al., 2019). A study showed that olanzapine downregulates the expression levels of mitochondrial fusion‐related mRNA (Scaini et al., 2018). Without causing toxicity in both *C. elegans* and cells (Figure S6), we observed the hyperfragmented mitochondrial network in both muscle and intestine cells of *C. elegans* exposed to olanzapine, which is consistent with previous data shown in L6 myoblast cells treated with olanzapine (Del Campo et al., 2018). These observations suggest that olanzapine may disrupt mitochondrial dynamics via the regulation of fusion‐related proteins. The remodeling of aberrant mitochondrial networks by UA treatments has been shown in previous studies (Kshirsagar et al., 2021a). This is consistent with our observations that UA ameliorated the hyperfragmented mitochondrial network in *C. elegans* exposed to olanzapine. Intriguingly, previous papers have confirmed that UA treatment can downregulate mitochondrial fission‐related genes and upregulate mitochondrial fusion‐related genes, which is essential to maintain mitochondrial function (Detmer & Chan, 2007; Kshirsagar et al., 2021b; Wu et al., 2022). Overall, improvement of the hyperfragmented mitochondrial network by UA in olanzapine‐treated nematodes may be modulated via mitochondrial dynamics, leading to the prevention of accelerated aging induced by olanzapine.

In conclusion, our data reveal the underlying mechanism of olanzapine‐accelerated aging is defective mitophagy. More specifically, olanzapine blocked the fusion between mitophagosomes and lysosomes, which can be restored by a mitophagy inducer, UA. This research opens a novel promising therapeutic strategy for preventing accelerated aging induced by olanzapine by using mitophagy inducers, such as UA.

## 4 MATERIALS AND METHODS

### 4.1 Reagents and antibodies

Olanzapine (11937), urolithin A (UA, 22607), tetramethylrhodamine ethyl ester (perchlorate) (TMRE, 21426) and, tetramethylrhodamine methyl ester (perchlorate) (TMRM, 21437) were purchased from Cayman chemical. Chloroquine diphosphate salt (CQ, C6628), sodium azide (NaN_3_, S8032), 5‐fluoro‐2′‐deoxyuridine (FUDR, F0503), 2‐butanone (360473), donkey serum (D9663), protease inhibitor cocktail (PIC, P8340), phenylmethanesulfonyl fluoride (PMSF, P7626), β‐glycerophosphate disodium salt hydrate (G9422), glucose (G8270), Triton X‐100 (X‐100), dimethyl sulfoxide (DMSO, 276855), carbenicillin disodium salt (C1389), isopropyl β‐D‐1‐thiogalactopyranoside (IPTG, I6758), ampicillin (A9393), and Amersham™ ECL western blotting detection reagent (RPN2106) were purchased from Sigma‐Aldrich. Mitotracker green FM (M7514), MitoSOX (M36008), MitoTracker Deep Red FM (M22426), ProLong™ Diamond Antifade Mountant (P36961), Lipofectamine™ 2000 transfection reagent (11668019), and NP40 cell lysis buffer (FNN0021) were purchased from Invitrogen. Mitochondria isolation kit for cultured cells (89874) was purchased from ThermoFisher Scientific. High‐Capacity cDNA Reverse Transcription Kit (4368814) was purchased from Applied Biosystems. Neurobasal medium (21103049), B‐27 supplement (17504044), L‐glutamine (25030149), and Penicillin–Streptomycin (Pen‐Strep, 15140122) were purchased from Gibco. Normocin (Ant‐nr‐1) was purchased from Invivogen. DC protein assay reagent kit (5000116) and Aurum™ Total RNA Mini Kit (7326820) were purchased from Bio‐Rad. Enhancing ATP Assay kit (S0027) was purchased from Beyotime. SensiFAST™ SYBR® No‐ROX Kit (BIO‐98020) was purchased from Meridian Bioscience. DNeasy Blood & Tissue Kit (69504) was purchased from Qiagen.

LC3B (D11) XP® Rabbit mAb (3868S), LAMP1 (D4O1S) Mouse mAb (15665S), PINK1 (D8G3) Rabbit mAb (6946S), Parkin (Prk8) Mouse mAb (4211S), AMPKα Antibody (2532S), Phospho‐AMPKα (Thr172) (40H9) Rabbit mAb (2535S), SQSTM1/P62 Antibody (5114S), LC3B Antibody (2775S), VDAC (D73D12) Rabbit mAb (4661S), COX IV Rabbit mAb (4850S), and Anti‐rabbit Antibody (7074S) were purchased from Cell Signaling Technology. Anti‐Actin Antibody (MAB1501), Anti‐mouse antibody (AP308P), and Anti‐MAP2 antibody (M4403) were purchased from Sigma‐Aldrich. Phalloidin‐iFluorTM 594 Conjugate (20553) was purchased from Cayman chemical. Alexa Fluor™ 488 Donkey anti‐Mouse Secondary Antibody (A‐21202), and Alexa Fluor™ 488 Donkey anti‐Rabbit Secondary Antibody (A‐21206) were purchased from Invitrogen.

### 4.2 Worm strains, maintenance, and pharmacological treatments

*C. elegans* strains were maintained on nematode growth media (NGM) agar plates seeded with OP50 *E. coli* strain (Caenorhabditis Genetics Center; CGC) at 20°C (Brenner, 1974). The following strains were obtained from CGC, which is funded by NIH Office of Research Infrastructure Programs (P40 OD010440): N2 Bristol wildtype, BZ555 *egIs1 [dat‐1p::GFP]*, SJ4143 *zcIs17 [ges‐1::GFP(mit)]*, SJ4103 *zcIs14 [myo‐3::GFP(mit)]*, MAH508 *sqEx67 [rgef‐1p::mCherry::GFP::lgg‐1 + rol‐6], egIs1 [dat‐1p::GFP]*, and SJZ42 *foxEx3 [rgef‐1p::tomm‐20::Rosella]*.

Olanzapine, UA, and CQ were added into the NGM at indicated concentrations just before pouring the plate. Worms were exposed to compounds during their whole life from the L4 larval stage until death unless stated otherwise. To ensure permanent exposure to compounds, plates were changed every 2–3 days. The experimental plate was stored at 4°C and used within 2 weeks. Olanzapine and UA were dissolved in DMSO as a stock solution, while CQ was freshly prepared by dissolving the powder in MilliQ water. The population was treated with the corresponding concentration of DMSO with the vehicle group as 0.1% at the final concentration.

The method for synchronization of *C. elegans* was performed according to standard protocols (Porta‐de‐la‐Riva et al., 2012; Stiernagle, 2006). A small scale of age‐synchronized worms was obtained from gravid worms, which laid eggs on agar plates for approximately 4–6 h until the required number of eggs had been laid. A high yield of synchronized worms was obtained by using the alkaline hypochlorite solution (bleach) for egg retrieval. When eggs hatched and developed into the L4 larval stage, age‐synchronized worms were used for further experiments.

### 4.3 Lifespan analysis of *C. elegans*

Lifespan examinations were performed on NGM agar plates seeded with *E. coli* OP50 lawn. The L4 larval stage (Day 0 of lifespan assay) worms were transferred onto NGM plates with or without olanzapine and UA. The first day of adulthood was recorded as Day 1 of lifespan. Animals were transferred into fresh plates every second day during the reproductive period and thereafter transferred twice a week. The number of worms was monitored every day, and any worm with no pharyngeal pumping or response to touch was considered dead. Worms that displayed internal hatching, exploded vulva, or ones that crawled off the plate were censored. Three independent experiments were conducted for each condition.

To minimize the confounding effect of bacterial metabolism on lifespan, the metabolically inactive OP50 was prepared according to the previous paper (Beydoun et al., 2021). The OP50 liquid culture mixed with PFA was incubated at 37°C for 1 h at the final concentration of 0.5%. After being washed with fresh LB four times, the OP50 was concentrated and seeded on NGM plates.

### 4.4 Health span analysis of *C. elegans*

The pharyngeal pumping rate was recorded by counting the number of contractions in the terminal bulb of the pharynx in 30 s intervals. For each time point, three independent experiments were carried out. For each independent experiment, 20 worms were used per condition.

The thrashing assay was performed by placing the worm in a drop of M9 buffer and allowing it to recover for 30 s (Huang et al., 2021). The number of body bends was manually counted and recorded at 30 s intervals. Twenty worms were examined per condition in three independent experiments.

The pathology of the deteriorated pharynx was measured as previously described (Ezcurra et al., 2018; Garigan et al., 2002). Worms were mounted onto 2% agar pads and anesthetized with 10 mM NaN_3_ in M9 buffer. The bright‐field channel pictures of the pharynx were taken with a DMi8 inverted fluorescence microscope (Leica Microsystems) using 20× objective lens. The phenotype of the pharynx was scored on an ordinal scale with scores 1–5, while 1 representing healthy and youthful appearance, 2 representing subtle deteriorated signs (uneven borders or small vacuoles inside intrapharyngeal bulb), 3 representing mild deteriorated phenotypes (expended lumen, swollen terminal bulb, or intrapharyngeal bulb cavities), 4 representing well‐developed deteriorated phenotypes shown at least two aforementioned aging features, and 5 representing extremely deteriorated appearance (swollen to the maximum level and barely recognizable bulb). The percentage of pharyngeal deterioration was calculated in total worms. For each time point, a minimum of 20 worms were measured.

Lipofuscin autofluorescence was determined by placing worms onto 2% agar pads and anesthetized with 10 mM NaN_3_, followed by DAPI channel images taken with a DMi8 inverted fluorescence microscope using 20× objective lens. The fluorescence intensity was measured by ImageJ (NIH). A minimum of 20 worms per condition were used.

### 4.5 Olfactory‐associated learning and memory assay of *C. elegans*

The olfactory‐associated learning and memory assay was performed as described in the Supplemental Experimental Procedures.

### 4.6 Detection of mitochondrial parameters in *C. elegans*

For detection of mitochondrial content, mitochondrial membrane potential, and mitochondrial ROS level, age‐synchronized worms at Day 9 of adulthood were cultured in NGM plates containing 125 nM Mitotracker green, 125 nM TMRE, and 10 μM MitoSOX at 20°C for 24 h. Next, worms were washed, placed onto 2% agar pads, and anesthetized with 10 mM NaN_3_. Images were taken with a DMi8 inverted fluorescence microscope under 20× objective lens and fluorescence intensity was analyzed using ImageJ. Twenty worms were examined per condition in two independent experiments.

### 4.7 Detection of mitochondrial network in *C. elegans*

Transgenic worms expressing GFP targeted to the outer mitochondrial membrane in their body wall muscle cells and intestine cells were used to detect the morphology of the mitochondrial network as previously described (Hartsough et al., 2020; Weir et al., 2017). Details are provided in the Supplemental Experimental Procedures.

### 4.8 Detection of mitophagy and autophagy in *C. elegans*

The mitophagy reporter strain *foxEx3 [rgef‐1p::tomm‐20::Rosella]* and autophagy reporter strain *sqEx67 [rgef‐1p::mCherry::GFP::lgg‐1 + rol‐6]* were used to examine the mitophagic flux and autophagic flux in the pan‐neuronal system of *C. elegans* as previously described (Chang et al., 2017; Cummins et al., 2019). Details are provided in the Supplemental Experimental Procedures.

### 4.9 Mammalian cell cultures and treatment

The cell line used in this study is HEK293T (CRL‐3216, ATCC), and HeLa‐Difluo™ hLC3 (Heldf‐hlc3, Invivogen). The HEK293T cells were cultured in Dulbecco's modified Eagle medium (DMEM) with 10% heat‐inactivated FBS and 2 mM L‐glutamine. The HeLa‐Difluo™ hLC3 Cells were cultured in DMEM with 10% heat‐inactivated FBS, 4.5 g/L glucose, 100 μg/mL Normocin, 100 U/mL Pen‐Strep. All cells were maintained at 37°C in an incubator with 5% CO_2_ and 95% relative humidity. Olanzapine, UA, and CQ were prepared as previously mentioned.

### 4.10 Analysis of mitochondrial parameters in HEK293T cells

After being exposed to compounds for 24 h, cells were incubated with different dyes, such as 40 nM TMRM for 15 min, 50 nM MitoTracker Green for 30 min, and 3 μM MitoSOX for 30 min, to detect the mitochondrial membrane potential, mitochondrial content, and mitochondrial ROS level, respectively. After being washed with PBS three times, cells were collected and analyzed by BD Accuri™ C6 Plus flow cytometer (BD Biosciences) and FlowJo software (BD).

### 4.11 Immunofluorescence assay

The immunofluorescence assay was described in the Supplemental Experimental Procedure.

### 4.12 Detection of mitophagic flux and autophagic flux in mammalian cells

The plasmid pCLBW COX8‐EGFP‐mCherry (78520, Addgene, David Chan) was used to detect mitophagic flux in mammalian cells. After HEK293T cells were seeded in plates containing coverslips for 24 h, transfection was carried out by using Lipofectamine™ 2000 transfection reagent, according to the manufacturer's protocol. Briefly, cells were treated with the reagent mixture of Lipofectamine 2000 and COX8‐EGFP‐mCherry plasmid for 6 h at 37°C, then the medium was replaced with the fresh complete culture medium. The next day, cells were exposed to compounds for another 24 h. After the cells were fixed and mounted with ProLong Diamond Antifade Mountant, the GFP and mCherry fluorescent signal inside the cell were taken with a TCS SP8 confocal laser scanning microscope using 63× oil immersion objective lens. The percentage of cells undergoing mitophagy was measured as the number of cells with mCherry‐only puncta in the number of total cells in each representative image, which was manually counted. The number of mCherry‐only puncta, representing mitolysosomes, inside each cell was manually counted. For detection of autophagic flux, HeLa‐Difluo™ hLC3 Cells were imaged by using the IncuCyte S3 live‐cell imaging platform and IncuCyte S3 software (Essen Bioscience) after treatments. The number of puncta shown as RFP‐positive (autolysosomes) or either GFP‐RFP‐positive (autophagosomes) was manually counted per cell. For each condition, 20 cells were analyzed.

### 4.13 Immunoblot analysis

The immunoblot analysis was described in the Supplemental Experimental Procedure.

### 4.14 Statistical analysis

Data are presented as mean ± SEM, unless otherwise specified. Student's *t* test was used for a two‐group comparison. One‐way analysis of variance (ANOVA) was used for three or more group comparisons followed by post hoc Tukey's multiple comparisons. Two‐way ANOVA followed by Bonferroni's multiple comparisons test was used for two categorical group comparisons. Differences in pharyngeal deterioration were analyzed using the Wilcoxon rank sum test. A chi‐squared test was used to compare the distribution of mitochondrial morphology into different categories. Survival analysis was performed using the Kaplan–Meier method and the significance of differences between survival curves was analyzed by the log‐rank test. All statistical analysis was carried out using GraphPad Prism 7.0 software (GraphPad Software Inc.) or SPSS Statistics 27.0 software (IBM Corporation). All *p* values <0.05 were considered to be statistically significant. ***p < 0.001, **p < 0.01, *p < 0.05, N.S., not significant.

## AUTHOR CONTRIBUTIONS

X. Chen, Y. Yu, and X. Huang designed experiments. X. Chen, Z. Wang, and X. Ge performed treatments and behavior tests of *C. elegans*. X. Chen, Z. Wang, P. Zheng, A. Dongol, and Y. Xie collected animal tissues and performed primary cortical neuron culture. X. Chen, M. Zheng, and X. Dang carried out cellular experiments. X. Chen and X. Huang wrote the manuscript, and all authors contributed to critical revisions of the final manuscript.

## FUNDING INFORMATION

This work was supported by funds from the National Health and Medical Research Council of Australia (NHMRC) awarded to Dr. X. Huang (Grant No.: APP1176503).

## CONFLICT OF INTEREST STATEMENT

No potential conflict of interest was reported by the author(s).

## Supporting information


Appendix S1

Figure S1

Figure S2

Figure S3

Figure S4

Figure S5

Figure S6

## Data Availability

The data used to support findings of this study are available from the corresponding author upon reasonable request.
