# Supplementary material for: Impaired mitophagosome–lysosome fusion mediates olanzapine‐induced aging
Source: Aging Cell. 2023 Oct 13;22(11):e14003. doi: 10.1111/acel.14003 (PMC10652317; doi:10.1111/acel.14003)
Supplement: Supplementary file 1 — Appendix S1 [file ACEL-22-e14003-s005.docx]

**Supplemental experimental procedures**

## RNA interference in *C. elegans*

RNAi experiment was performed by feeding with HT115 bacteria carrying L4440 vector expressing the dsRNA against the gene of interest or empty L4440 vector as the negative control. RNAi clones were obtained from the Ahringer Library (Garigan et al., 2002; Kamath & Ahringer, 2003). RNAi-feeding bacteria were grown overnight at 37°C in Luria broth (LB) with 100 μg/ml ampicillin. The RNAi feeding plate was prepared with the NGM plate supplemented with 25 µg/ml carbenicillin and 1 mM IPTG. Eggs were placed on RNAi feeding plates to induce the dsRNA expression for the lifespan assay.

## Olfactory-associated learning and memory assay of *C. elegans*

The Olfactory-associated learning and memory assay was carried out as previously described (Fenyves et al., 2021; Kauffman et al., 2011). In brief, age-synchronized worms were exposed to compounds, and cultured in NGM plates containing 100 µM FUDR to prevent reproduction. At day 1 or 5 of adulthood, the attraction to the chemoattractant butanone was tested by using the chemotaxis assay. The chemotaxis assay was performed on the 100 mm NGM plates without seeding the bacterial lawn. At least 50 worms were placed on an original marked spot and given 1 h to choose another spot of either 10% butanone dissolved in ethanol with 1M NaN_3_, or a reference spot of ethanol with NaN_3_ at room temperature. After 1 h, the number of worms within a 1 cm radius of either an attractant, reference spot, or original marked spot, as well as the total number of worms were manually counted. The chemotaxis index (CI) was calculated as $CI=\left( n_{\mathrm{Butanone}}-n_{\mathrm{EtOH}} \right)/\left( n_{\mathrm{Total}}-n_{\mathrm{Origin}} \right)$. The age-synchronized worms were exposed to starvation for 1 h, then the chemotaxis assay (Naïve) was performed. After a 1 h short-term training session by seeding 10% butanone on the inside of the lid of a 60 mm NGM plate with bacterial lawn, the chemotaxis assay (conditioned) was performed and the short-term learning index (STLI) was calculated as SML$I=\mathrm{CI}_{\mathrm{conditioned}}-\mathrm{CI}_{Naïve}$. Following 1 h rest in the NGM plate with bacterial lawn, but no butanone, worms were collected for the chemotaxis assay (1 h delay) and the short-term memory index (STMI) was calculated as $STMI=\mathrm{CI}_{1 h delay}-\mathrm{CI}_{Naïve}$. For the long-term training, starved worms were repeatedly exposed to NGM plates with butanone for 1 h and then starved for another 30 min. After being conditioned in NGM plates with butanone 7 times, worms were performed the chemotaxis assay, and the long-term leaning index (LTLI) was calculated as $LTLI=\mathrm{CI}_{\mathrm{conditioned}}-\mathrm{CI}_{Naïve}$. After worms were incubated in NGM plates with bacterial lawn for 24 h, the chemotaxis assay was performed (24 h delay), and the long-term memory index (LTMI) was calculated as $LTMI=\mathrm{CI}_{24 h delay}-\mathrm{CI}_{Naïve}$. At least 3 independent test plates were used per condition.

## Egg laying assay

The fecundity was examined by using L4-stage synchronized worms. The nematode was transferred onto individual NGM agar plates seeded with OP50 and allowed to lay eggs at 20°C for 24 h. Thereafter, each worm was transferred to new plates on the following day until day 5 of adulthood. Offspring were maintained at 20°C, and the number of progeny was counted 2 days later. Twenty worms were examined per condition in 3 independent experiments.

## Dopaminergic neurodegeneration assay in C. elegans

The degeneration of dopaminergic neurons was conducted by using transgenic worm BZ555 expressing GFP driven by dopaminergic-specific promoter *dat-1* as previously described (Beilina et al., 2020). Age-synchronized worms were placed onto 2% agar pads and anesthetized with 10 mM NaN3. The image of four cephalic (CEP) dopaminergic neurons was taken by TCS SP5 confocal laser scanning microscope with a 63X oil-immersion objective lens. The percentage of normal surviving neurons with degenerative features, such as blebbed, broken, or absent axons, as well as absent or fragmented cell soma, were calculated in total worms. A minimum of 15 worms were analyzed per condition at different time points.

## Detection of mitochondrial network in *C. elegans* (continued)

Worms were exposed to compounds and placed onto 2% agar pads at day 9 of adulthood. After worms were anesthetized with 10 mM NaN3, images of the mitochondrial network in body wall muscle cells and intestine cells were taken using a DMi8 inverted fluorescence microscope and TCS SP5 confocal laser scanning microscope (Leica, Germany), respectively, with 63X oil-immersion objective lenses. The mitochondrial network for each cell was classified as either tubular (highly connective), intermediate (containing regions of both types), or fragmented (isolated and circular clusters of fluorescence). The percentage of cells showing tubular, fused, or fragmented mitochondrial networks in the total number of cells was used for analysis. A minimum of 8 muscle or intestine cells from at least 8 worms were analyzed per condition.

## Detection of mitophagy and autophagy in *C. elegans* (continued)

Worms were exposed to different compounds from L4 larval stage until day 5 of adulthood. After being placed onto 2% agar pads and anesthetized with 10 mM NaN3, an image of anterior head neurons for each worm was taken by a TCS SP5 confocal laser scanning microscope with 63X oil-immersion objective lens. For each condition, 20 worms were analyzed. For analysis of mitophagic activity, the fluorescence intensity of both GFP and DsRed for each worm was measured by ImageJ, and the GFP/DsRed ratio was calculated as the mitophagy index. For analysis of autophagy, the number of mCherry-only puncta (autophagosome) and the total number of puncta (positive in both mCherry and GFP channels, autolysosome) were calculated.

## Immunofluorescence assay

After seeding onto coverslips and exposing to compounds for 24 h, HEK293T cells were stained with Mitotracker Deep Red at a final concentration of 50 nM for 30 min. Cells were then washed again with PBS, and fixed with 4% paraformaldehyde for 15 min at room temperature. Then, cells were permeabilized with 0.3% Triton X-100 in PBS and blocked with 5% donkey serum in PBS for 1h at room temperature. Thereafter, cells were incubated with primary antibodies, such as LC3B antibody (1:3000) and LAMP1 antibody (1:100), in 1% donkey serum at 4°C overnight. On the following day, cells were washed with PBS and incubated with Alexa-conjugated secondary antibodies (1:400) in 1% donkey serum for 1h at room temperature. Finally, cells were washed with PBS, and coverslipped glass microscope slides containing a drop of ProLong Diamond Antifade Mountant upside down. Images were taken with a TCS SP8 confocal laser scanning microscope using 63X oil-immersion objective lens. The co-localization between mitochondria and autophagosomes or lysosomes was measured by manually counting the number of puncta shown positive in both mitotracker and LC3 or either mitotracker and LAMP1, respectively.

## Immunoblot analysis

After treatments, cells were harvested and lysed with lysis buffer comprised of PIC (100X), 1 mM PMSF, and 0.5 mM β-glycerophosphate dissolved in NP40 buffer. Following centrifugation at 5000 RPM for 5 min, the supernatant was collected for detection of protein concentration, which was measured using a DC protein assay reagent kit and SpectraMax Plus 384 Microplate Reader (Molecular Devices, USA), according to manufacturer's instructions. Then, 20 µg of protein was mixed with laemmli buffer and denatured at 95°C for 5 min. Thereafter, samples were loaded onto 26-well gels (5671095, Bio-Rad), and electrophoresed at 120 V for 90 min at room temperature. After SDS-PAGE, gels were transferred into Amersham™ Hybond® P western blotting membranes (E10600021, Merck) at 100 V for 1 h at 4°C. Membranes were blocked with 5% skim milk for 1 h at room temperature and incubated with primary antibodies at 4°C overnight, with PINK1 antibody (1:1000), Parkin antibody (1:1000), AMPKα antibody (1:1000), Phospho-AMPKα antibody (1:1000), SQSTM1/P62 antibody (1:1000), LC3B antibody (1:1000), VDAC antibody (1:1000), COX IV antibody (1:1000) and Anti-Actin antibody (1:3000). Furthermore, membranes were incubated with HRP-conjugated secondary antibodies for 1 h at room temperature, with either anti-rabbit antibody (1:3000) or an anti-mouse antibody (1:3000). Immunoreactivity was detected by using Amersham™ ECL western blotting detection reagent. Images were captured by using Amersham Imager 600 RGB (GE Health, Chicago, USA), while the quantification was performed with ImageJ.

## Subcellular fraction

The mitochondrial fraction and cytosolic fraction were separated by using the Mitochondrial Isolation Kit according to the manufacturer’s instructions. The isolated mitochondrial pellet was resuspended in the RIPA buffer supplemented with the proteinase inhibitor cocktail on ice for 1 h, and the extracted protein was analyzed by immunoblot as described above.

## Determination of adenosine triphosphate (ATP) content

The ATP content was determined by using the Enhancing ATP Assay kit, according to the manufacturer’s instructions. The ATP content was normalized against the total protein concentration.

## MTT assay

*In vitro* cytotoxicity was determined by MTT assay as previously described (Wang et al., 2023).

## Quantitative real-time PCR (qRT-PCR) for gene expression and mtDNA/nDNA ratio

The total RNA was extracted with Aurum™ Total RNA Mini Kit, according to the manufacturer’s instructions. The cDNA was prepared with the High-Capacity cDNA Reverse Transcription Kit following the manufacturer’s instructions. The total DNA for cells and *C. elegans* were extracted with DNeasy Blood & Tissue Kit, according to the manufacturer’s instructions. The RT-PCR reaction was performed using SensiFAST™ SYBR® No-ROX Kit and the QuantStudio 5 Real-Time PCR System (Applied Biosystems) with the primers listed in Table S2. The fold change of gene expression was calculated with the comparative ΔΔ*Ct* method, and normalized to the vehicle group. Three biological replicates were done for each group.

## Primary cortical neuron culture

Primary cortical neurons were prepared as previously described (Hilgenberg & Smith, 2007). Cortical neurons were harvested from the postnatal day 0-3 of C57BL/6J mice, which has been approved by the Animal Ethics Committee (AE 19/15), University of Wollongong, Australia, and complied with the Australian Code of Practice for the Care and Use of Animals for Scientific Purposes. Briefly, dissociated cortical neurons were collected after the cortex had been trypsinized for 30 min and then the supernatant was centrifuged at 100 *g* for 5 min. Cortical neurons were seeded in plates containing coverslips, which had been coated with poly-D-lysine (Sigma-Aldrich, P6407) at 4°C overnight, and cultured in Neurobasal medium with B-27 supplement, 100 U/ml Pen-Strep, and 0.1 M glucose at 37°C in an incubator with 5% CO_2_ and 95% relative humidity. After 24 h of culture, 10 μM FUDR was added into the culture medium to avoid the growth of non-neuronal cells.

## Quantification of neurite outgrowth, sholl analysis, and determination of dendritic spine density

Cortical neurons were cultured *in vitro* for 7 days (DIV7), and treated with olanzapine for another 24 h. Then, cells were seeded onto the coverslip, fixed, permeabilized, and blocked as aforementioned. After being incubated with Anti-MAP2 antibody (1:800) at 4°C overnight, cells were incubated with Alexa Fluor 488-conjugated anti-mouse antibody for 1 h at room temperature. Thereafter, coverslips were mounted onto glass microscope slides and visualized under a TCS SP5 confocal laser scanning microscope with 63X oil-immersion objective lens. The total neurite length and sholl analysis for each cell were quantified by ImageJ software. Cortical neurons at DIV21 were treated with olanzapine for 24 h and used for the analysis of spine density. After fixation and permeabilization, cells were stained with Phalloidin-iFluorTM 594 Conjugate (1:1000) for 90 min at room temperature. Coverslips were mounted onto glass microscope slides and visualized under TCS SP5 confocal laser scanning microscope with 63X oil-immersion objective lens. The spine density was calculated by using the ImageJ software. Dendritic segments that were not overlapping with other branches were selected randomly for analysis, with the criteria of being 50 μm away from the soma. At least four dendritic segments per cell with a total neurite length of more than 80 μm were measured.

**Supplementary references**

Beilina, A., Bonet-Ponce, L., Kumaran, R., Kordich, J. J., Ishida, M., Mamais, A., Kaganovich, A., Saez-Atienzar, S., Gershlick, D. C., Roosen, D. A., Pellegrini, L., Malkov, V., Fell, M. J., Harvey, K., Bonifacino, J. S., Moore, D. J., & Cookson, M. R. (2020). The Parkinson's disease protein LRRK2 Interacts with the GARP complex to promote retrograde transport to the trans-Golgi network. *Cell Rep, 31*(5), 107614. doi:h
<ttps://doi.org/10.1016/j.celrep.2020.107614>

Fenyves, B. G., Arnold, A., Gharat, V. G., Haab, C., Tishinov, K., Peter, F., de Quervain, D., Papassotiropoulos, A., & Stetak, A. (2021). Dual role of an mps-2/KCNE-dependent pathway in long-term memory and age-dependent memory decline. *Current Biology, 31*(3), 527-539.e527. doi:h
<ttps://doi.org/10.1016/j.cub.2020.10.069>

Garigan, D., Hsu, A. L., Fraser, A. G., Kamath, R. S., Ahringer, J., & Kenyon, C. (2002). Genetic Analysis of Tissue Aging in Caenorhabditis elegans: A Role for Heat-Shock Factor and Bacterial Proliferation. *Genetics, 161*(3), 1101-1112. doi:h
<ttps://doi.org/10.1093/genetics/161.3.1101>

Gilliver, S. C., Ruckshanthi, J. P. D., Hardman, M. J., Nakayama, T., & Ashcroft, G. S. (2008). Sex Dimorphism in Wound Healing: The Roles of Sex Steroids and Macrophage Migration Inhibitory Factor. *Endocrinology, 149*(11), 5747-5757. doi:h
<ttps://doi.org/10.1210/en.2008-0355>

Hilgenberg, L. G., & Smith, M. A. (2007). Preparation of dissociated mouse cortical neuron cultures. *J Vis Exp*(10), 562. doi:h
<ttps://doi.org/10.3791/562>

Kamath, R. S., & Ahringer, J. (2003). Genome-wide RNAi screening in Caenorhabditis elegans. *Methods, 30*(4), 313-321. doi:h
<ttps://doi.org/10.1016/S1046-2023(03)00050-1>

Kauffman, A., Parsons, L., Stein, G., Wills, A., Kaletsky, R., & Murphy, C. (2011). C. elegans positive butanone learning, short-term, and long-term associative memory assays. *J Vis Exp*(49). doi:h
<ttps://doi.org/10.3791/2490>

Lin, Y. F., Schulz, A. M., Pellegrino, M. W., Lu, Y., Shaham, S., & Haynes, C. M. (2016). Maintenance and propagation of a deleterious mitochondrial genome by the mitochondrial unfolded protein response. *Nature, 533*(7603), 416-419. doi:h
<ttps://doi.org/10.1038/nature17989>

Qian, X., Li, X., Shi, Z., Bai, X., Xia, Y., Zheng, Y., Xu, D., Chen, F., You, Y., Fang, J., Hu, Z., Zhou, Q., & Lu, Z. (2019). KDM3A Senses Oxygen Availability to Regulate PGC-1α-Mediated Mitochondrial Biogenesis. *Mol Cell, 76*(6), 885-895.e887. doi:h
<ttps://doi.org/10.1016/j.molcel.2019.09.019>

Vandesompele, J., De Preter, K., Pattyn, F., Poppe, B., Van Roy, N., De Paepe, A., & Speleman, F. (2002). Accurate normalization of real-time quantitative RT-PCR data by geometric averaging of multiple internal control genes. *Genome Biol, 3*(7), Research0034. doi:h
<ttps://doi.org/10.1186/gb-2002-3-7-research0034>

Wang, Z., Zheng, P., Nagaratnam, N., Solowij, N., & Huang, X. F. (2023). Parkin Mediates Cannabidiol Prevention of Amyloid-Beta-Induced Senescence in Human Astrocytes. *Cannabis Cannabinoid Res, 8*(2), 309-320. doi:h
<ttps://doi.org/10.1089/can.2022.0186>

Zhang, Q., Wang, Z., Zhang, W., Wen, Q., Li, X., Zhou, J., Wu, X., Guo, Y., Liu, Y., Wei, C., Qian, W., & Tian, Y. (2021). The memory of neuronal mitochondrial stress is inherited transgenerationally via elevated mitochondrial DNA levels. *Nature Cell Biology, 23*(8), 870-880. doi:<https://doi.org/10.1038/s41556-021-00724-8>

| Experiments | | Strains | Bacterial | Groups | Number of worms | Median lifespan (day) | Mean ± SEM (day) | | Change in lifespan (%) | statistics (*p*-value) |
| --- | --- | --- | --- | --- | --- | --- | --- | --- | --- | --- |
| Exp.1 | 1^st^ repeat | N2 | OP50 | Vehicle | 97 | 15.00 | 16.31 | 0.52 |  |  |
|  |  |  |  | Olanzapine 25 µM | 95 | 14.00 | 13.98 | 0.46 | -14.29% | *p* = 0.0004 vs Vehicle |
|  |  |  |  | Olanzapine 50 µM | 94 | 15.00 | 14.53 | 0.43 | -10.90% | *p* = 0.0009 vs Vehicle |
|  |  |  |  | Olanzapine 100 µM | 95 | 12.00 | 12.53 | 0.34 | -23.20% | *p* < 0.0001 vs Vehicle |
|  |  |  |  | Olanzapine 150 µM | 81 | 12.00 | 12.11 | 0.26 | -25.74% | *p* < 0.0001 vs Vehicle |
|  |  |  |  | Olanzapine 300 µM | 87 | 12.00 | 11.76 | 0.36 | -27.90% | *p* < 0.0001 vs Vehicle |
|  | 2^nd^ repeat |  |  | Vehicle | 125 | 16.00 | 15.39 | 0.29 |  |  |
|  |  |  |  | Olanzapine 25 µM | 100 | 13.00 | 13.08 | 0.23 | -15.02% | *p* < 0.0001 vs Vehicle |
|  |  |  |  | Olanzapine 50 µM | 123 | 13.00 | 13.11 | 0.19 | -14.80% | *p* < 0.0001 vs Vehicle |
|  |  |  |  | Olanzapine 100 µM | 123 | 12.00 | 12.40 | 0.25 | -19.45% | *p* < 0.0001 vs Vehicle |
|  |  |  |  | Olanzapine 150 µM | 131 | 13.00 | 13.01 | 0.22 | -15.49% | *p* < 0.0001 vs Vehicle |
|  |  |  |  | Olanzapine 300 µM | 152 | 13.00 | 13.42 | 0.22 | -12.81% | *p* < 0.0001 vs Vehicle |
|  | 3^rd^ repeat |  |  | Vehicle | 132 | 15.00 | 15.30 | 0.40 |  |  |
|  |  |  |  | Olanzapine 25 µM | 87 | 12.00 | 12.72 | 0.31 | -16.81% | *p* < 0.0001 vs Vehicle |
|  |  |  |  | Olanzapine 50 µM | 94 | 11.00 | 11.46 | 0.29 | -25.09% | *p* < 0.0001 vs Vehicle |
|  |  |  |  | Olanzapine 100 µM | 92 | 11.00 | 11.35 | 0.27 | -25.81% | *p* < 0.0001 vs Vehicle |
|  |  |  |  | Olanzapine 150 µM | 147 | 12.00 | 12.53 | 0.22 | -18.08% | *p* < 0.0001 vs Vehicle |
|  |  |  |  | Olanzapine 300 µM | 95 | 12.00 | 12.16 | 0.28 | -20.51% | *p* < 0.0001 vs Vehicle |
| Exp.2 | 1^st^ repeat | N2 | OP50 | Vehicle | 138 | 13.00 | 13.48 | 0.37 |  |  |
|  |  |  |  | Olanzapine 150 µM | 127 | 11.00 | 11.47 | 0.30 | -14.92% | *p* < 0.0001 vs Vehicle |
|  |  |  |  | Urolithin A 50 µM | 111 | 17.00 | 17.70 | 0.39 | 31.28% | *p* < 0.0001 vs Vehicle |
|  |  |  |  | Olanzapine 150 µM + Urolithin A 50 µM | 110 | 13.00 | 12.63 | 0.27 | 10.13% | *p* = 0.0079 vs Olanzapine |
|  | 2^nd^ repeat |  |  | Vehicle | 93 | 15.00 | 15.60 | 0.53 |  |  |
|  |  |  |  | Olanzapine 150 µM | 113 | 10.00 | 10.22 | 0.18 | -34.52% | *p* < 0.0001 vs Vehicle |
|  |  |  |  | Urolithin A 50 µM | 113 | 17.00 | 19.28 | 0.57 | 23.59% | *p* < 0.0001 vs Vehicle |
|  |  |  |  | Olanzapine 150 µM + Urolithin A 50 µM | 103 | 12.00 | 12.24 | 0.20 | 19.80% | *p* < 0.0001 vs Olanzapine |

Table S1: Summary of the lifespan experiment in *C. elegans*.

| Experiments | | Strains | Bacterial | Groups | Number of worms | Median lifespan (day) | Mean ± SEM (day) | | Change in lifespan (%) | statistics (*p*-value) |
| --- | --- | --- | --- | --- | --- | --- | --- | --- | --- | --- |
| Exp.2 | 3^rd^ repeat | N2 | OP50 | Vehicle | 114 | 18.00 | 17.72 | 0.55 |  |  |
|  |  |  |  | Olanzapine 150 µM | 107 | 13.00 | 12.75 | 0.26 | -28.06% | *p* < 0.0001 vs Vehicle |
|  |  |  |  | Urolithin A 50 µM | 99 | 20.00 | 20.07 | 0.64 | 13.27% | *p* = 0.0113 vs Vehicle |
|  |  |  |  | Olanzapine 150 µM + Urolithin A 50 µM | 113 | 15.00 | 15.72 | 0.36 | 23.29% | *p* < 0.0001 vs Olanzapine |
| Exp.3 | 1^st^ repeat | N2 | HT115 | Vehicle | 72 | 20 | 19.89 | 0.61 |  | *p =* 0.0171 vs Vehicle |
|  |  |  | HT115 | Olanzapine 150 µM | 60 | 16 | 17.63 | 0.64 | -11.34% |  |
|  |  |  | *pink-1* | Vehicle | 56 | 20.00 | 20.02 | 0.61 |  | *p =* 0.5557 vs Vehicle |
|  |  |  | *pink-1* | Olanzapine 150 µM | 51 | 17.00 | 18.78 | 0.95 | -6.16% |  |
|  |  |  | *dct-1* | Vehicle | 64 | 20.00 | 19.61 | 0.60 |  | *p =* 0.0572 vs Vehicle |
|  |  |  | *dct-1* | Olanzapine 150 µM | 48 | 21.00 | 20.00 | 1.01 | 1.99% |  |
|  |  |  | *bec-1* | Vehicle | 62 | 14.00 | 14.44 | 0.36 |  | *p =* 0.2492 vs Vehicle |
|  |  |  | *bec-1* | Olanzapine 150 µM | 44 | 15.00 | 14.95 | 0.54 | 3.60% |  |
|  | 2^nd^ repeat | N2 | HT115 | Vehicle | 62 | 21.00 | 20.16 | 0.60 |  | *p* < 0.0001 vs Vehicle |
|  |  |  | HT115 | Olanzapine 150 µM | 69 | 16.00 | 17.07 | 0.50 | -15.32% |  |
|  |  |  | *pink-1* | Vehicle | 56 | 23.00 | 21.55 | 0.72 |  | *p =* 0.1867 vs Vehicle |
|  |  |  | *pink-1* | Olanzapine 150 µM | 55 | 19.00 | 19.35 | 0.81 | -10.25% |  |
|  |  |  | *dct-1* | Vehicle | 55 | 17.00 | 18.02 | 0.67 |  | *p =* 0.6344 vs Vehicle |
|  |  |  | *dct-1* | Olanzapine 150 µM | 53 | 16.00 | 17.26 | 0.80 | -4.18% |  |
|  |  |  | *bec-1* | Vehicle | 60 | 14.00 | 15.05 | 0.41 |  | *p =* 0.6376 vs Vehicle |
|  |  |  | *bec-1* | Olanzapine 150 µM | 50 | 15.00 | 15.28 | 0.50 | 1.53% |  |
|  | 3^rd^ repeat | N2 | HT115 | Vehicle | 68 | 20.00 | 19.02 | 0.60 |  | *p =* 0.0498 vs Vehicle |
|  |  |  | HT115 | Olanzapine 150 µM | 56 | 15.00 | 16.82 | 0.69 | -11.54% |  |
|  |  |  | *pink-1* | Vehicle | 59 | 21.00 | 20.34 | 0.63 |  | *p =* 0.3154 vs Vehicle |
|  |  |  | *pink-1* | Olanzapine 150 µM | 51 | 15.00 | 17.69 | 0.87 | -13.04% |  |
|  |  |  | *dct-1* | Vehicle | 61 | 17.00 | 18.59 | 0.68 |  | *p =* 0.6854 vs Vehicle |
|  |  |  | *dct-1* | Olanzapine 150 µM | 52 | 17.00 | 18.69 | 0.83 | 0.55% |  |
|  |  |  | *bec-1* | Vehicle | 68 | 15.00 | 15.29 | 0.41 |  | *p =* 0.3982 vs Vehicle |
|  |  |  | *bec-1* | Olanzapine 150 µM | 56 | 15.00 | 15.71 | 0.52 | 2.75% |  |

| Experiments | | Strains | Bacterial | Groups | Number of worms | Median lifespan (day) | Mean ± SEM (day) | | Change in lifespan (%) | statistics (*p*-value) |
| --- | --- | --- | --- | --- | --- | --- | --- | --- | --- | --- |
| Exp.4 | 1^st^ repeat | N2 | PFA-killed OP50 | Vehicle | 40 | 19 | 19.13 | 0.69 |  | *p =* 0.0057 vs Vehicle |
|  |  |  |  | Olanzapine 150 µM | 51 | 15 | 16.65 | 0.50 | -12.96% |  |
|  | 2^nd^ repeat |  |  | Vehicle | 40 | 18 | 19.18 | 0.67 |  | *p =* 0.0389 vs Vehicle |
|  |  |  |  | Olanzapine 150 µM | 70 | 15 | 16.81 | 0.56 | -12.31% |  |
|  | 3^rd^ repeat |  |  | Vehicle | 47 | 19 | 19.11 | 0.61 |  | *p =* 0.0099 vs Vehicle |
|  |  |  |  | Olanzapine 150 µM | 50 | 16 | 16.56 | 0.60 | -13.33% |  |

Table S2: The primer used in the paper

| Primer name | Sequence (5’-3’) | Source |
| --- | --- | --- |
| PGC-1α-F | GGACATGTGCAACCAGGACTC | (Qian et al., 2019) |
| PGC-1α-R | GAAGATCTGGGCAAAGAGGCT |  |
| TFAM-F | CCAAAAAGACCTCGTTCAGCTT |  |
| TFAM-R | CTTCAGCTTTTCCTGCGGTG |  |
| GAPDH-F | TGCACCACCAACTGCTTAGC | (Gilliver et al., 2008; Vandesompele et al., 2002) |
| GAPDH-R | GGCATGGACTGTGGTCATGAG |  |
| mtDNA-F in cells | CACCCAAGAACAGGGTTTGT | (Zhang et al., 2021) |
| mtDNA-R in cells | TGGCCATGGGTGTGTTGTTA |  |
| nDNA-F in cells | CAAAACCTAACTTGCGCAGA |  |
| nDNA-R in cells | TTTTAGGATGGCAAGGGACT |  |
| mtDNA-F in *C. elegans* | GCTTTTTCTTTATATGTTTTGTG | (Lin et al., 2016) |
| mtDNA-R in *C. elegans* | TCACCTTCAGAAAAATCAAATGG |  |
| nDNA-F in *C. elegans* | AGGCTAAGCCGGGGTAAGTT |  |
| nDNA-R in *C. elegans* | GCCAAAAGCTTAAACTGCGG |  |

Uncropped WB membrane for Figure 2d

150

100

50

20

1

37

150


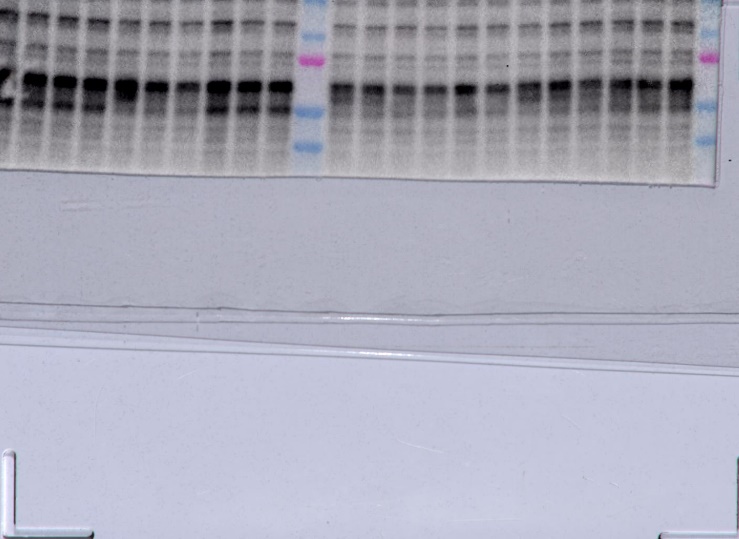


F-PINK1

C-PINK1

50

75

100

150

250

100

75

50


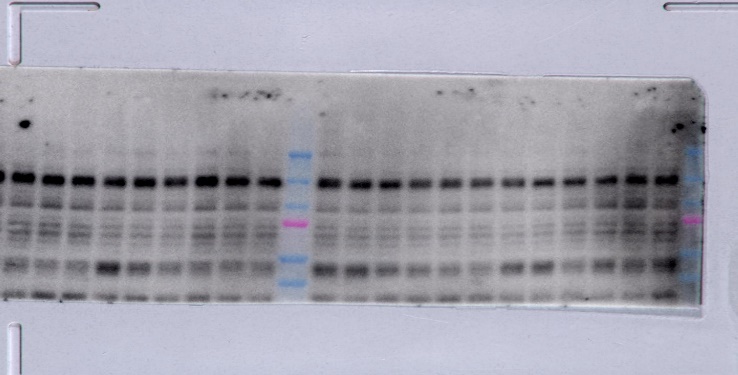


Parkin

37


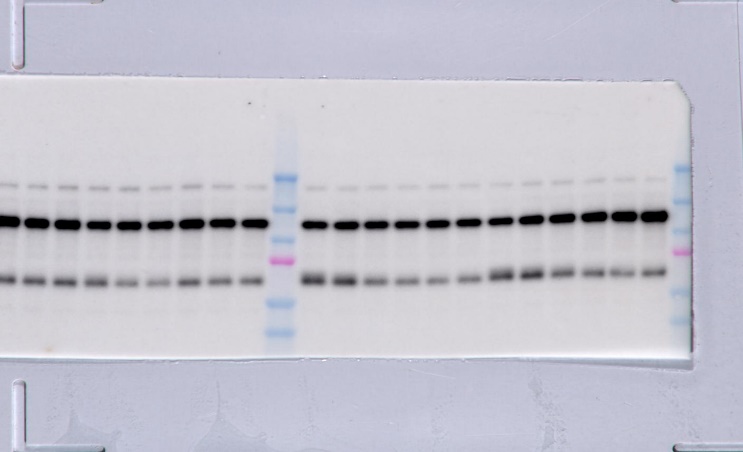


50

37

75

100

150

250

*p*-AMPK


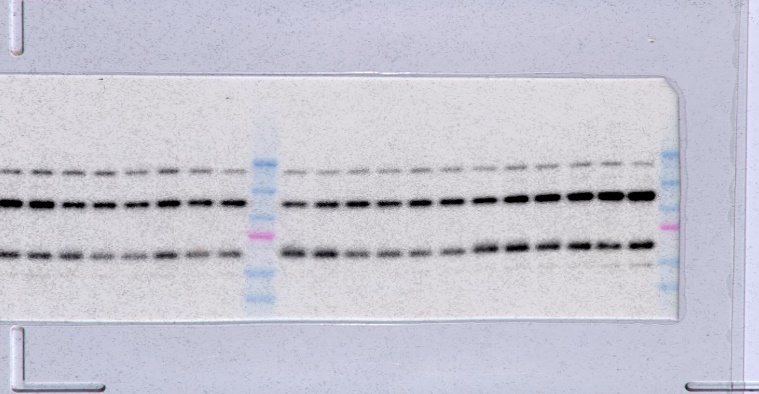


AMPK

150

250

100

75

50

37


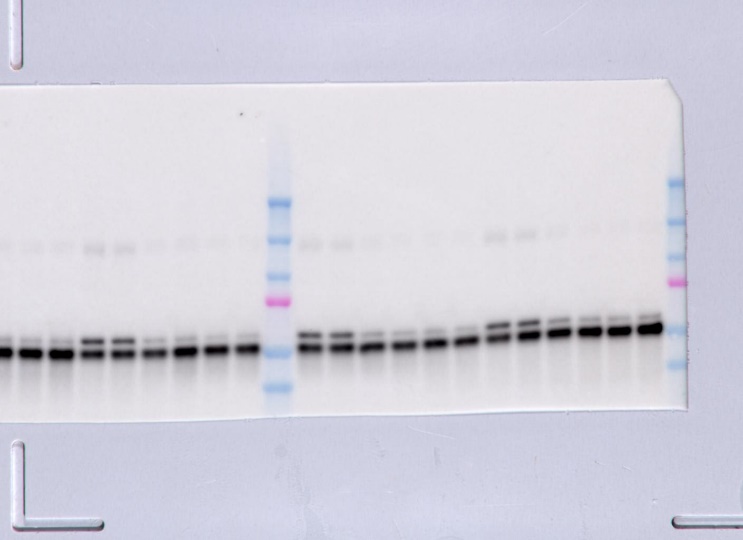


SQST-1/P62

37

50

75

100

150

250


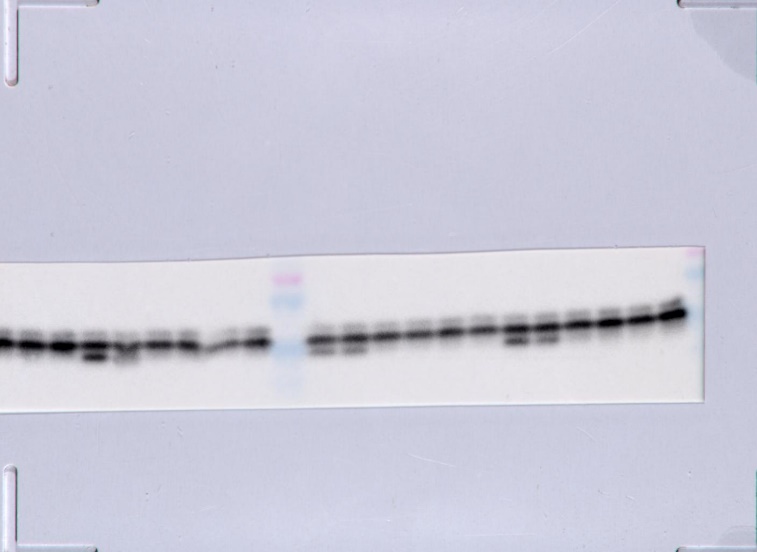


LC3-I

LC3-II

15

20

25

50


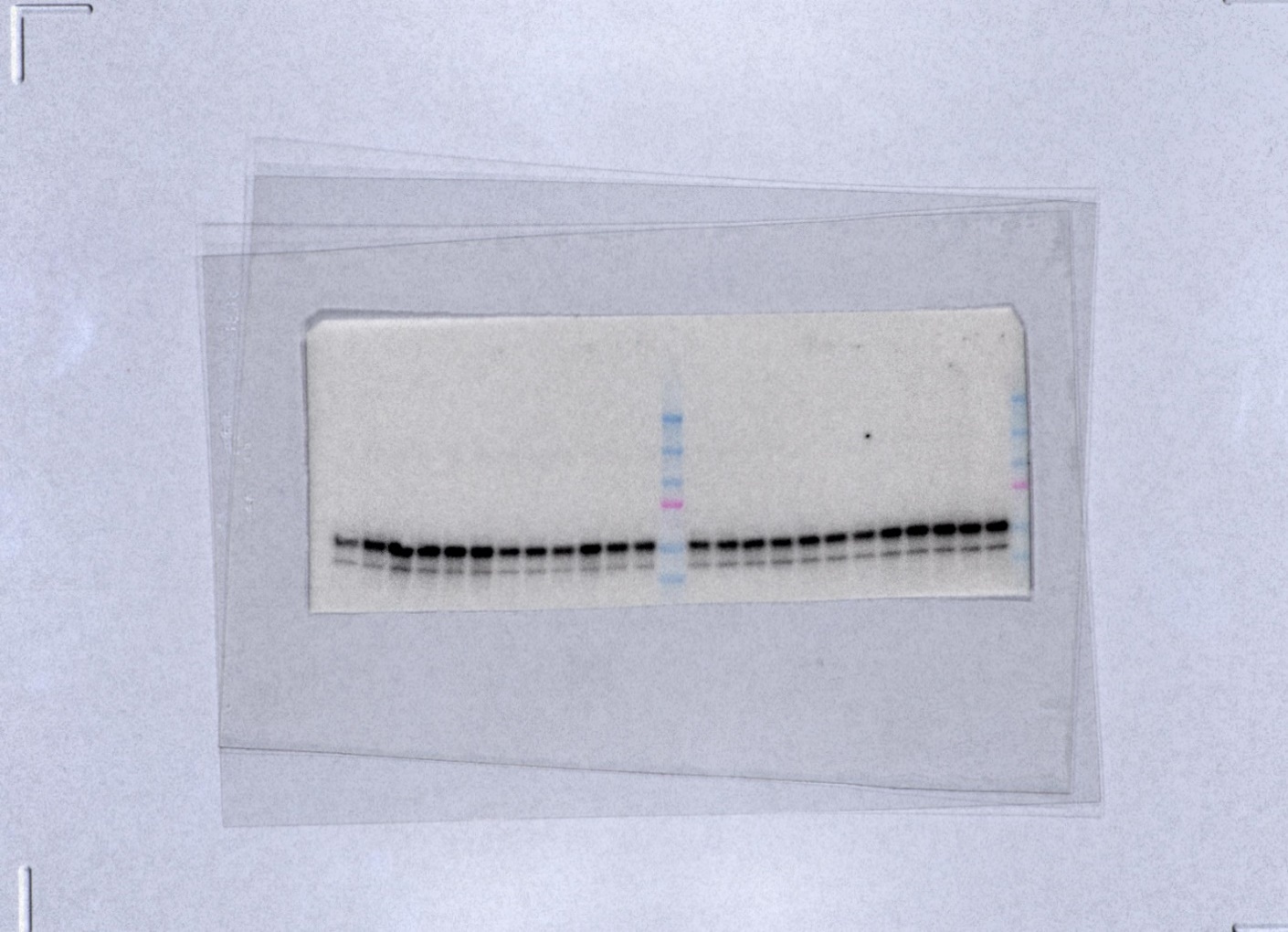


75

37

100

150

250

Actin

Olan (μM)

0

0

1

20

50

100

150

kDa

Uncropped WB membrane for Figure 5a

kDa

150

100

75

250

50

37

25

20

15

10

UA


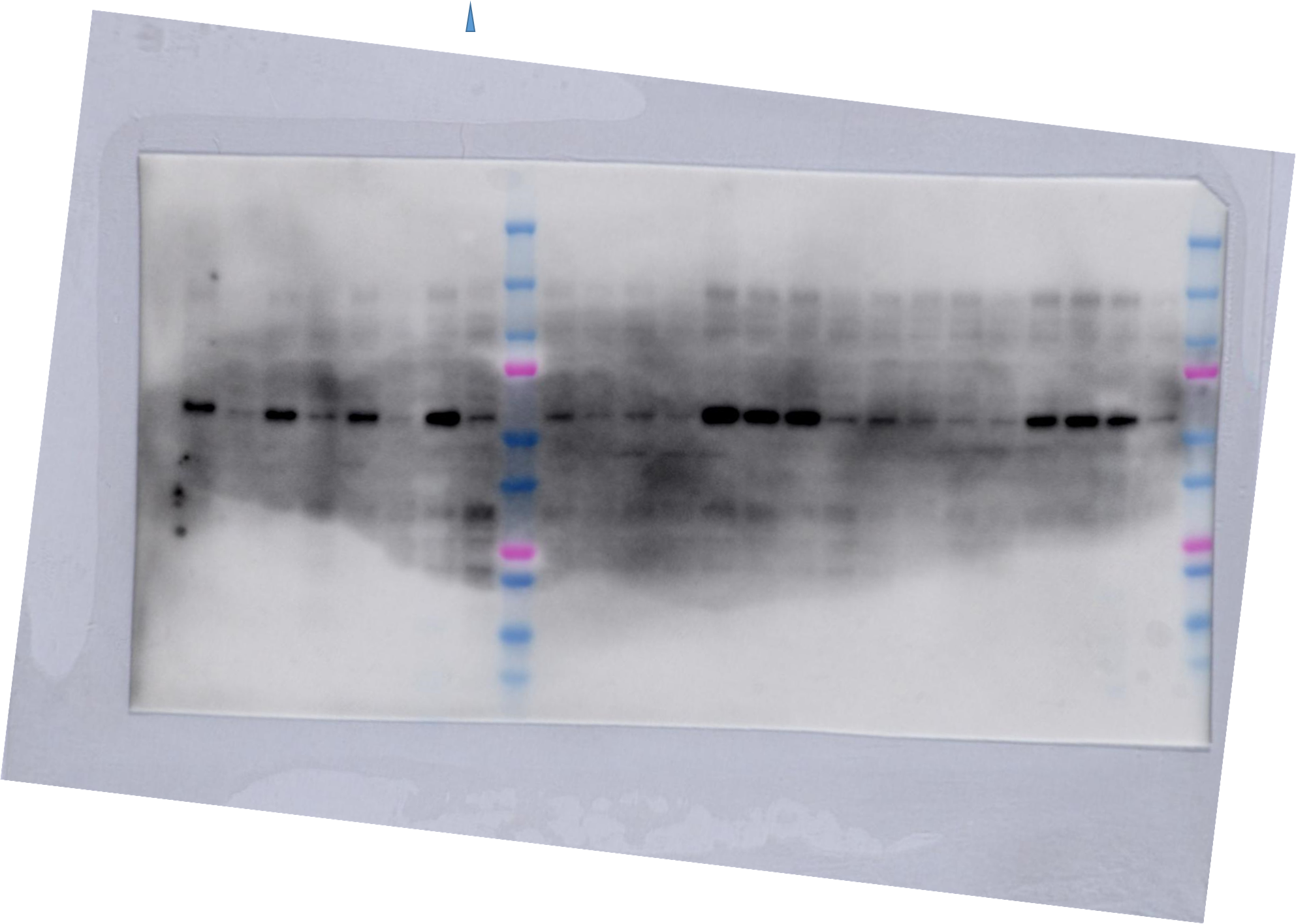


SQST-1/P62


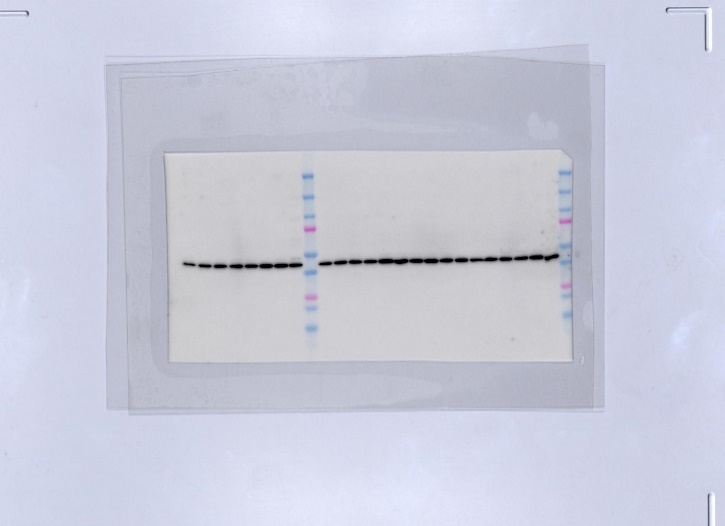


Actin

Vehicle

Olan

Olan+UA

Vehicle

Olan

UA

Olan+UA

Olan

Olan+UA


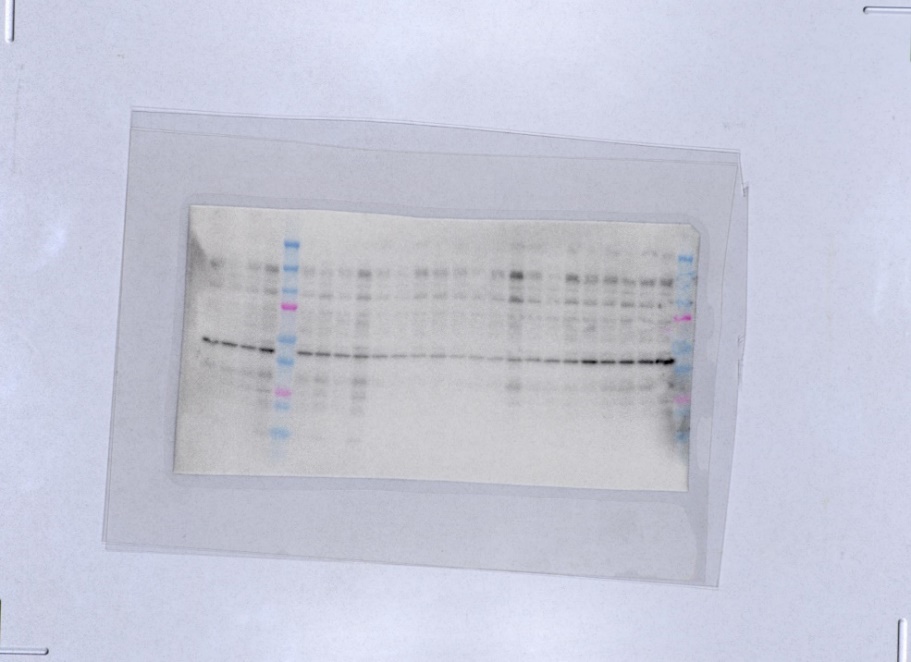


Actin


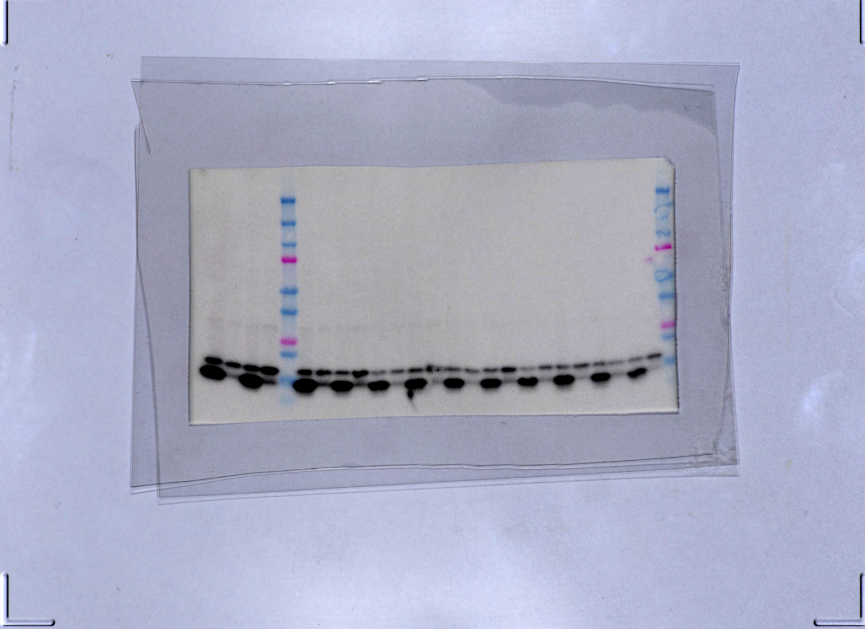


LC3-I

LC3-II

Vehicle

UA

Vehicle

Olan

UA

Olan+UA

kDa

150

100

75

250

50

37

25

20

15

10

kDa

150

100

75

250

50

37

25

20

15

10

kDa

150

100

75

250

50

37

25

20

10

Uncropped WB membrane for Figure 5b

UA

Olan + UA

Vehicle

Olan

Olan + BafA

Olan + CQ

Olan + UA + BafA


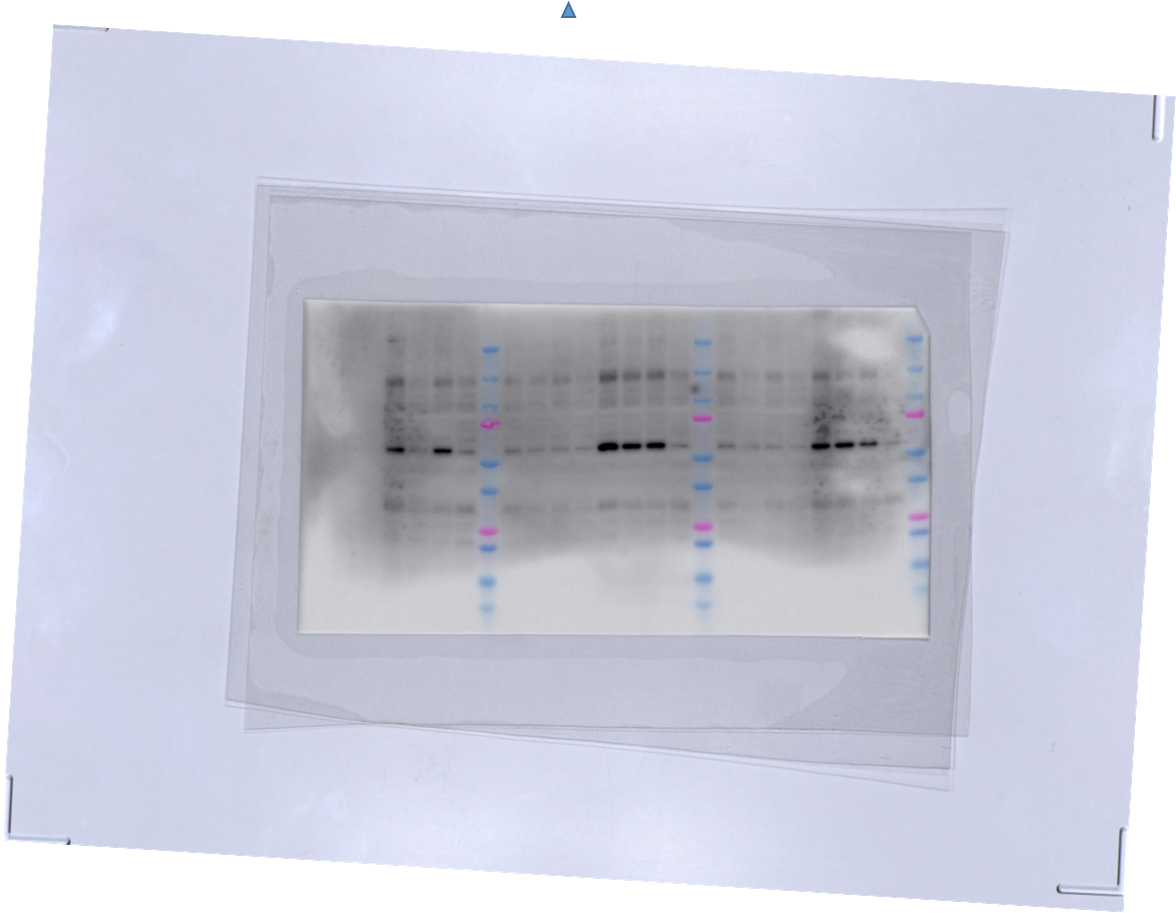

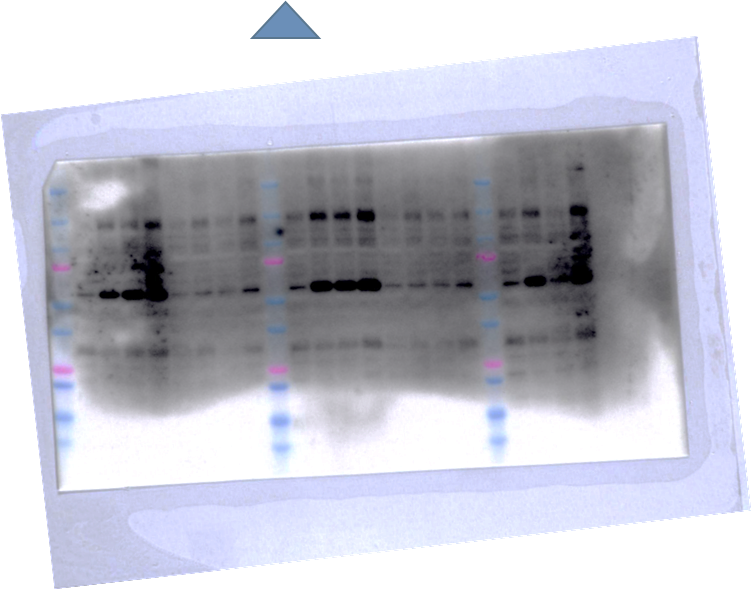


SQST-1/P62 (short exposure)

SQST-1/P62 (long exposure)

Olan + UA + CQ


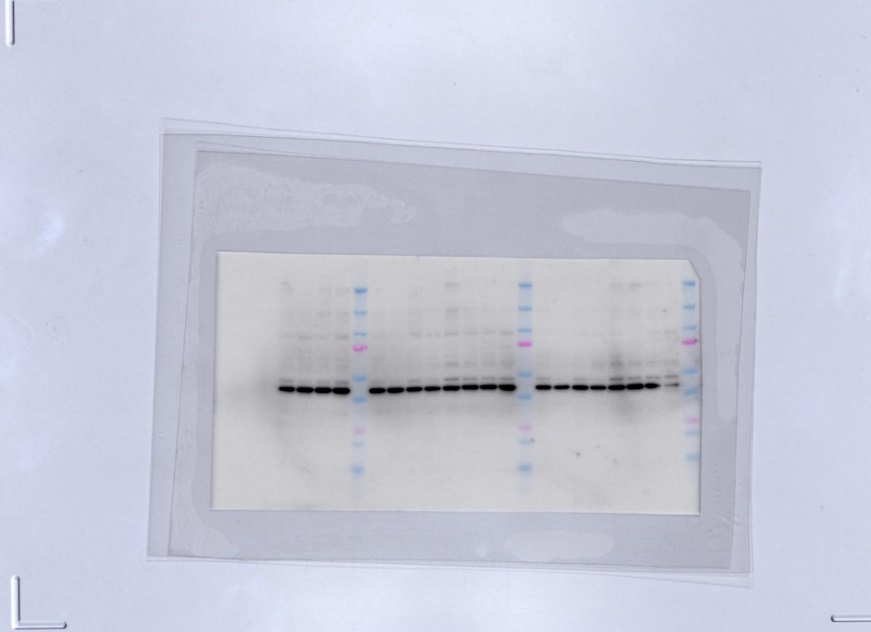


Actin

kDa

150

100

75

250

50

37

25

20

10

15

kDa

150

100

75

250

50

37

25

20

10

15

kDa

150

100

75

250

50

37

25

20

10

15


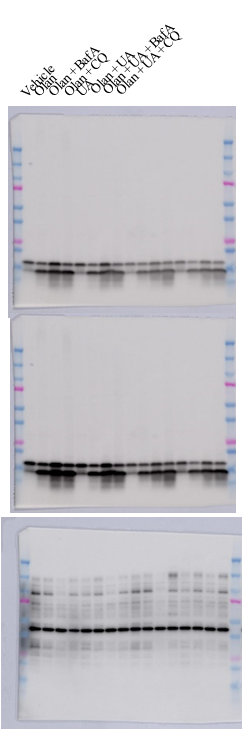


UA

Olan + UA

Vehicle

Olan

Olan + BafA

Olan + CQ

Olan + UA + BafA

Olan + UA + CQ

LC3-I

LC3-II

(short exposure)

LC3-I

LC3-II

(long exposure)

Actin

kDa

150

100

75

250

50

37

25

20

10

15

kDa

150

100

75

250

50

37

25

20

10

15

kDa

150

100

75

250

50

37

25

20

10

15

uncropped WB membrane for supplementary figure S2d


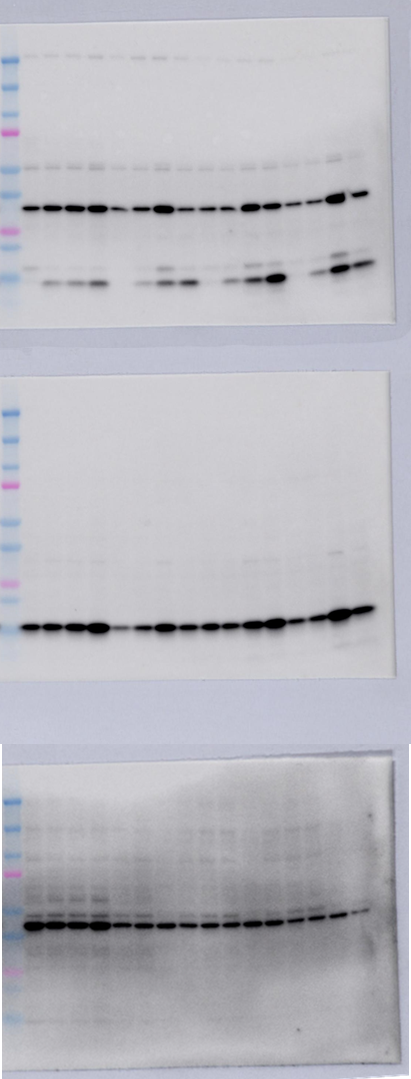


Olan

Vehicle

Actin

COX IV

VDAC

kDa

150

100

75

250

50

37

25

20

10

15

kDa

150

100

75

250

50

37

25

20

10

15

kDa

150

100

75

250

50

37

25

20

10

15

uncropped WB membrane for supplementary figure S3a

SQST-1/P62


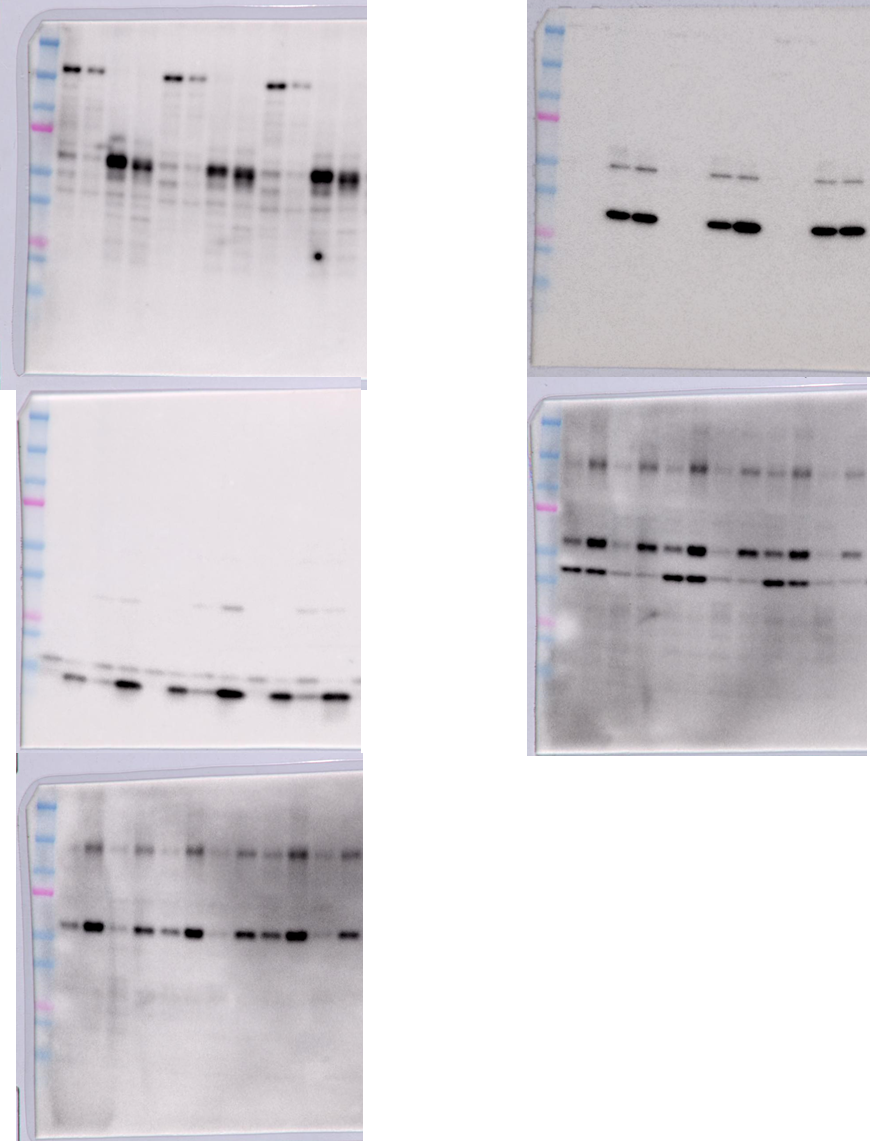


Cytosol

Mito

Olan

Veh

Olan

Veh

Cytosol

Mito

Olan

Veh

Olan

Veh

F-PINK1

C-PINK1

LC3-I

LC3-II

VDAC

Actin

kDa

150

100

75

250

50

37

25

20

10

15

kDa

150

100

75

250

50

37

25

20

10

15

kDa

150

100

75

250

50

37

25

20

10

15

kDa

150

100

75

250

50

37

25

20

10

15

kDa

150

100

75

250

50

37

25

20

10

15

uncropped WB membrane for supplementary figure S3b

Olan+ CQ

Olan+BafA

Vehicle

Actin

LC3-II

LC3-I

SQST-1/P62

(long exposure)

(Short exposure)


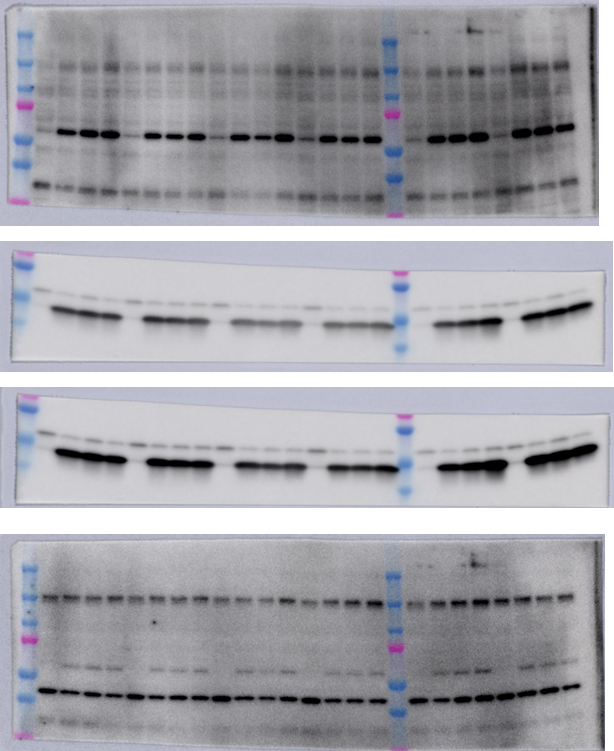


LC3-I

LC3-II

Olan

kDa

150

100

75

250

50

37

25

25

20

10

15

kDa

25

20

10

kDa

15

kDa

150

100

75

250

50

37

25

uncropped WB membrane for supplementary figure S5a


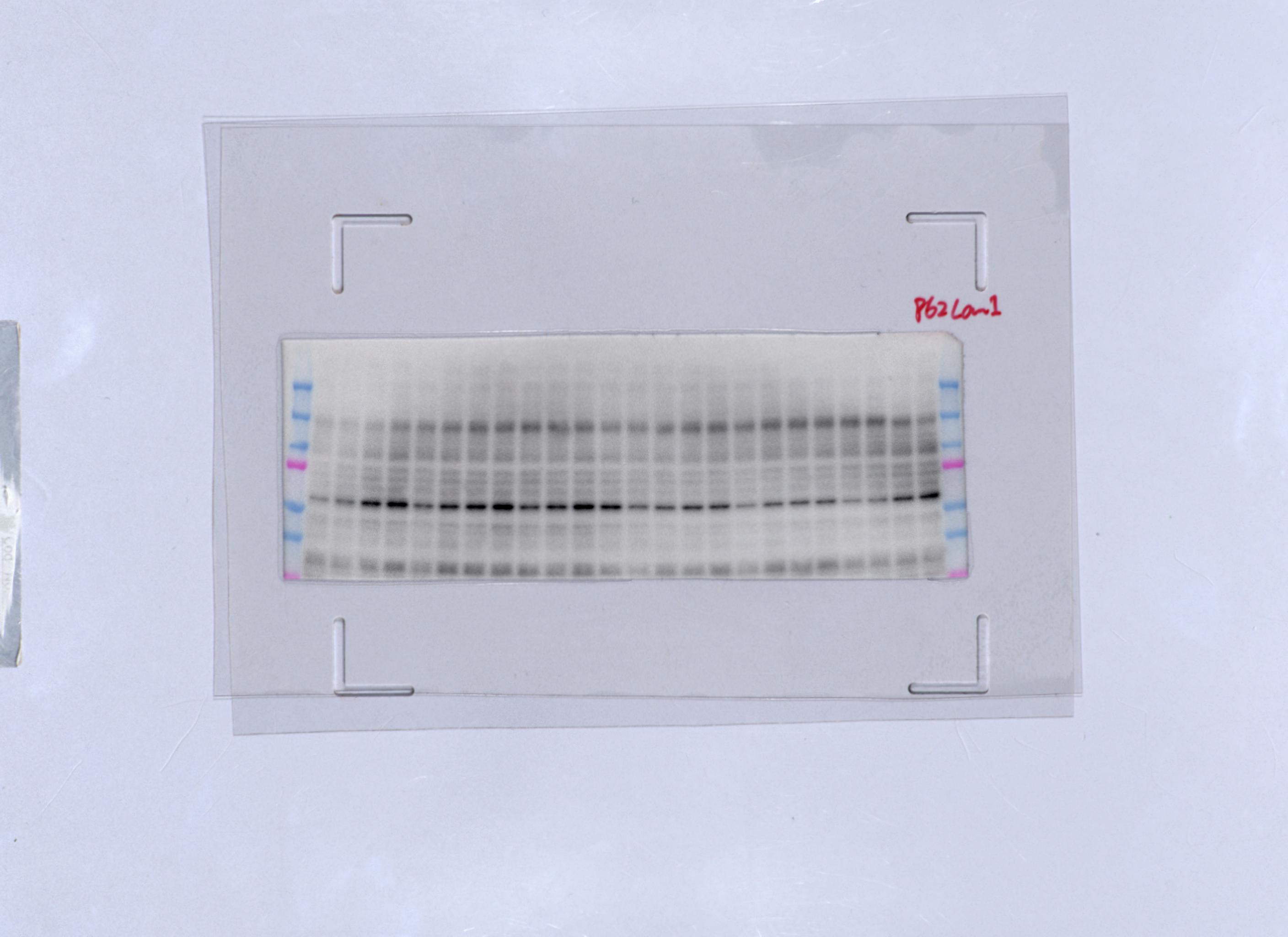

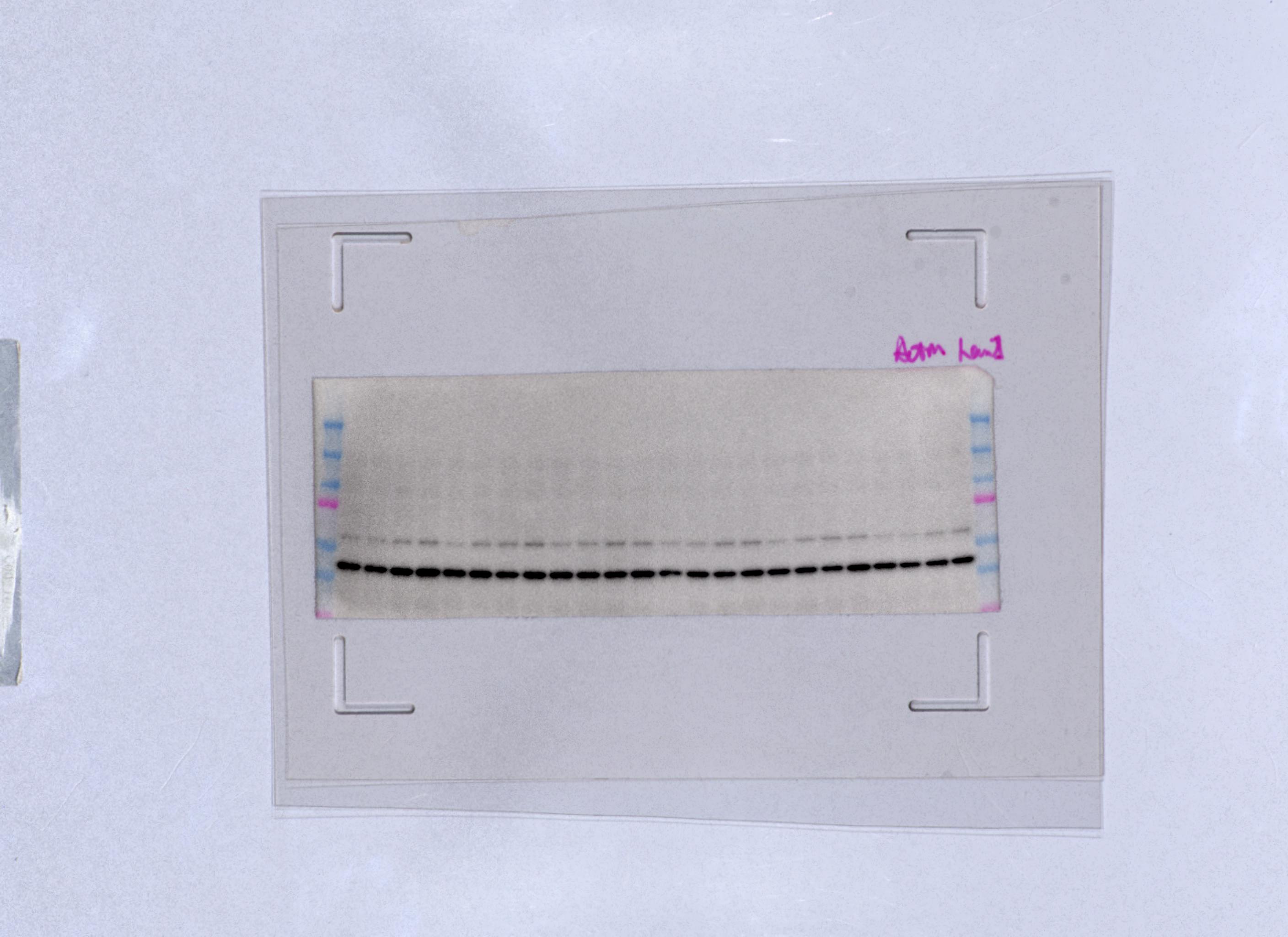

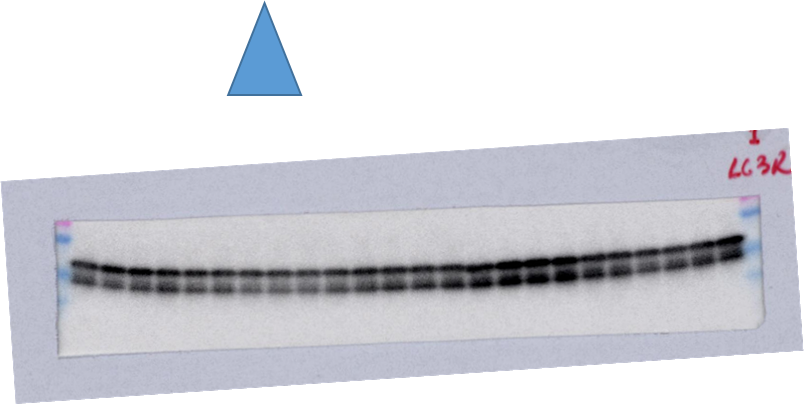


SQST-1/P62

Actin

LC3-II

LC3-I

kDa

150

100

75

250

50

37

25

25

20

10

15

kDa

kDa

150

100

75

250

50

37

25
