# Supplementary figures and images for: Impaired mitophagosome–lysosome fusion mediates olanzapine‐induced aging
Source: Aging Cell. 2023 Oct 13;22(11):e14003. doi: 10.1111/acel.14003 (PMC10652317; doi:10.1111/acel.14003)

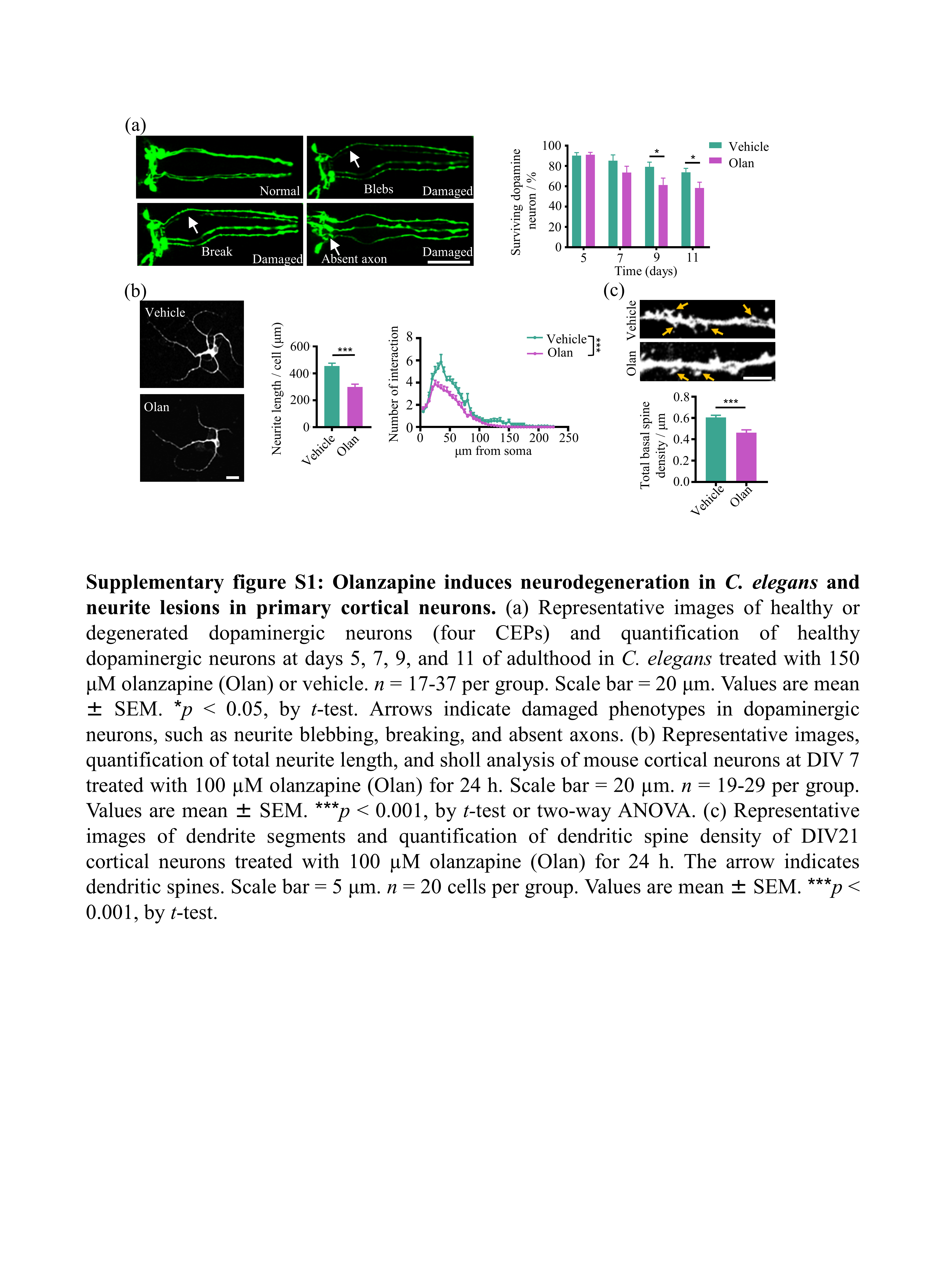

Supplement: Supplementary file 2 — Figure S1 [file ACEL-22-e14003-s003.tiff]

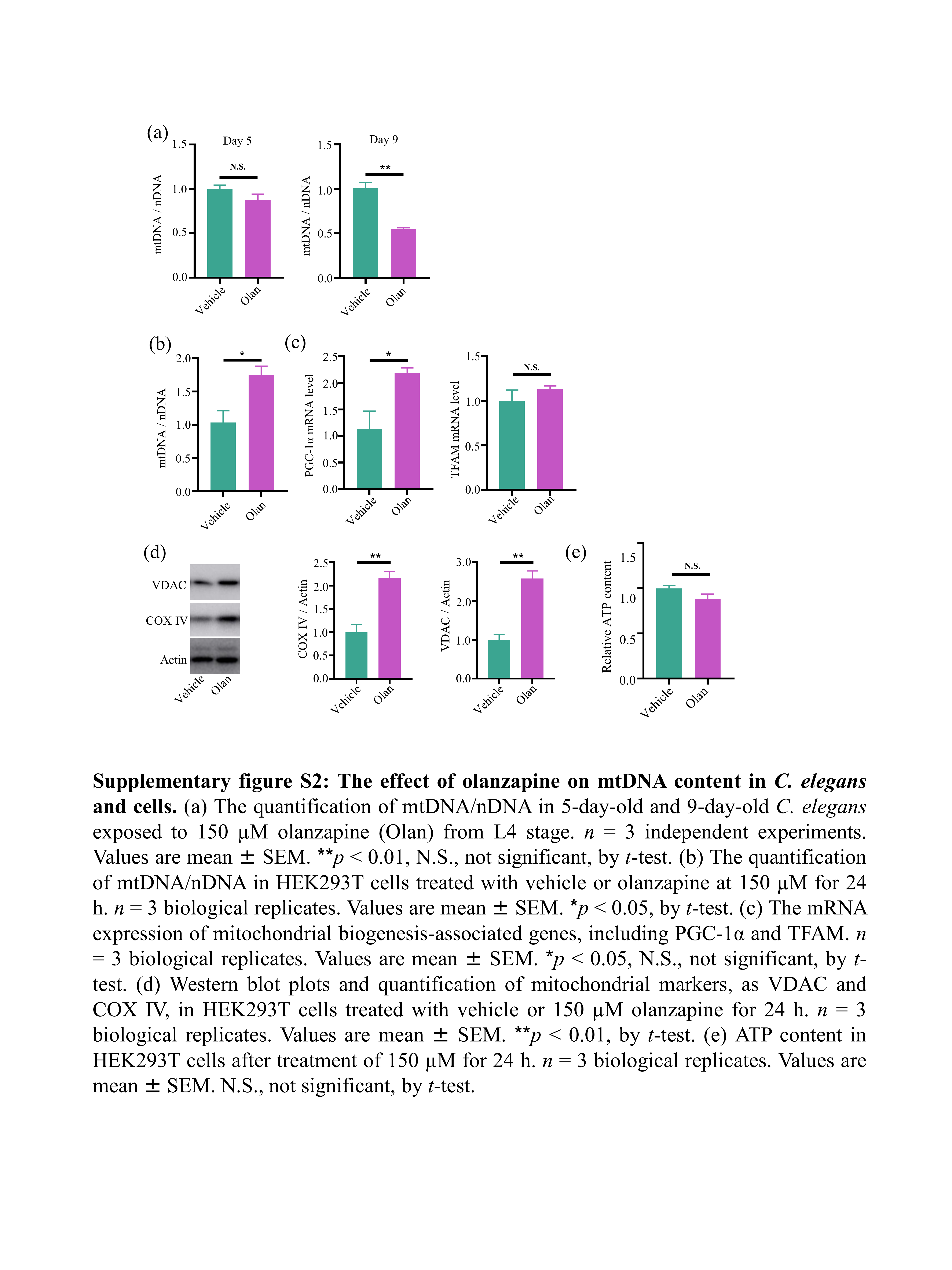

Supplement: Supplementary file 3 — Figure S2 [file ACEL-22-e14003-s002.tiff]

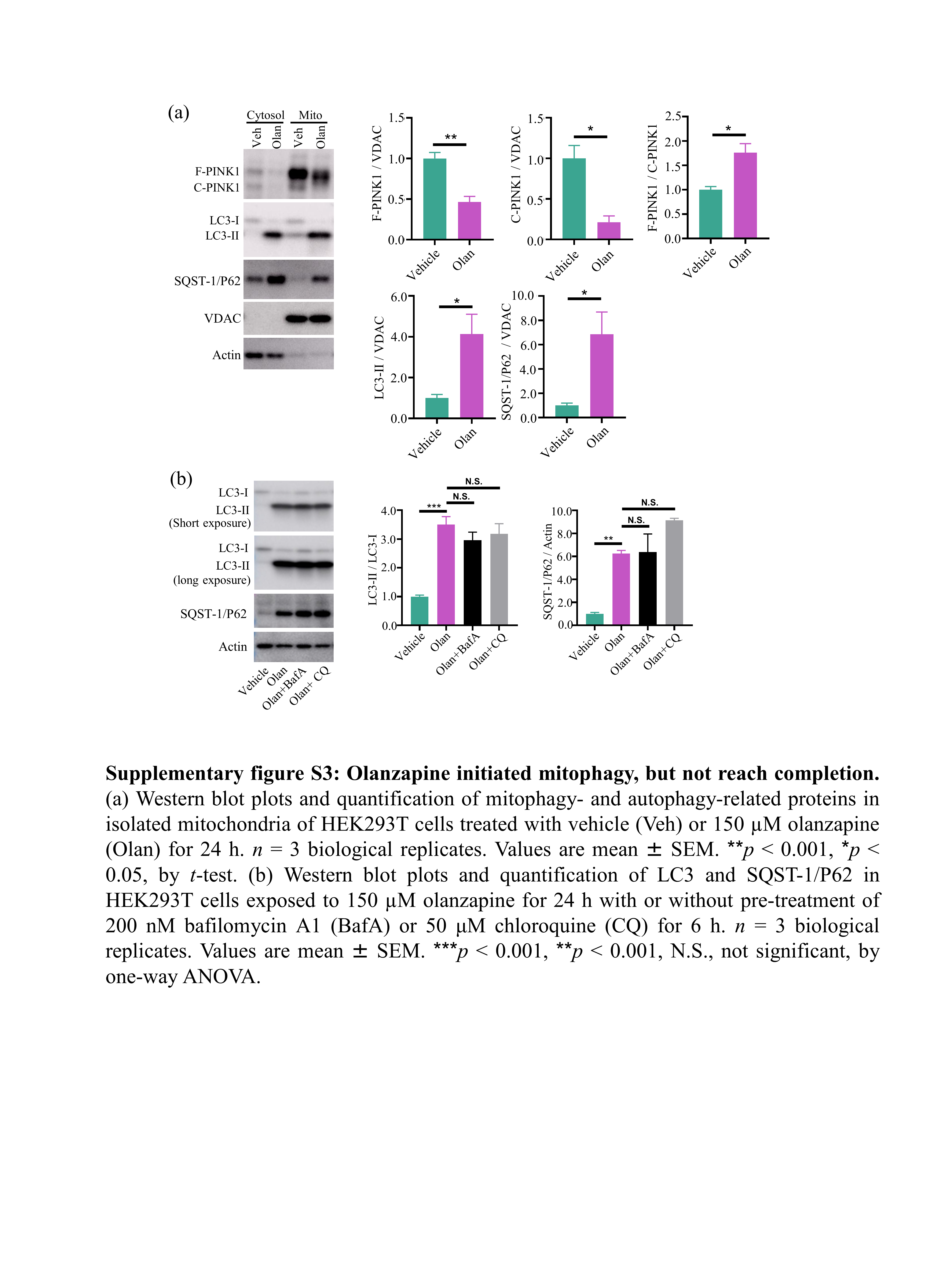

Supplement: Supplementary file 4 — Figure S3 [file ACEL-22-e14003-s006.tiff]

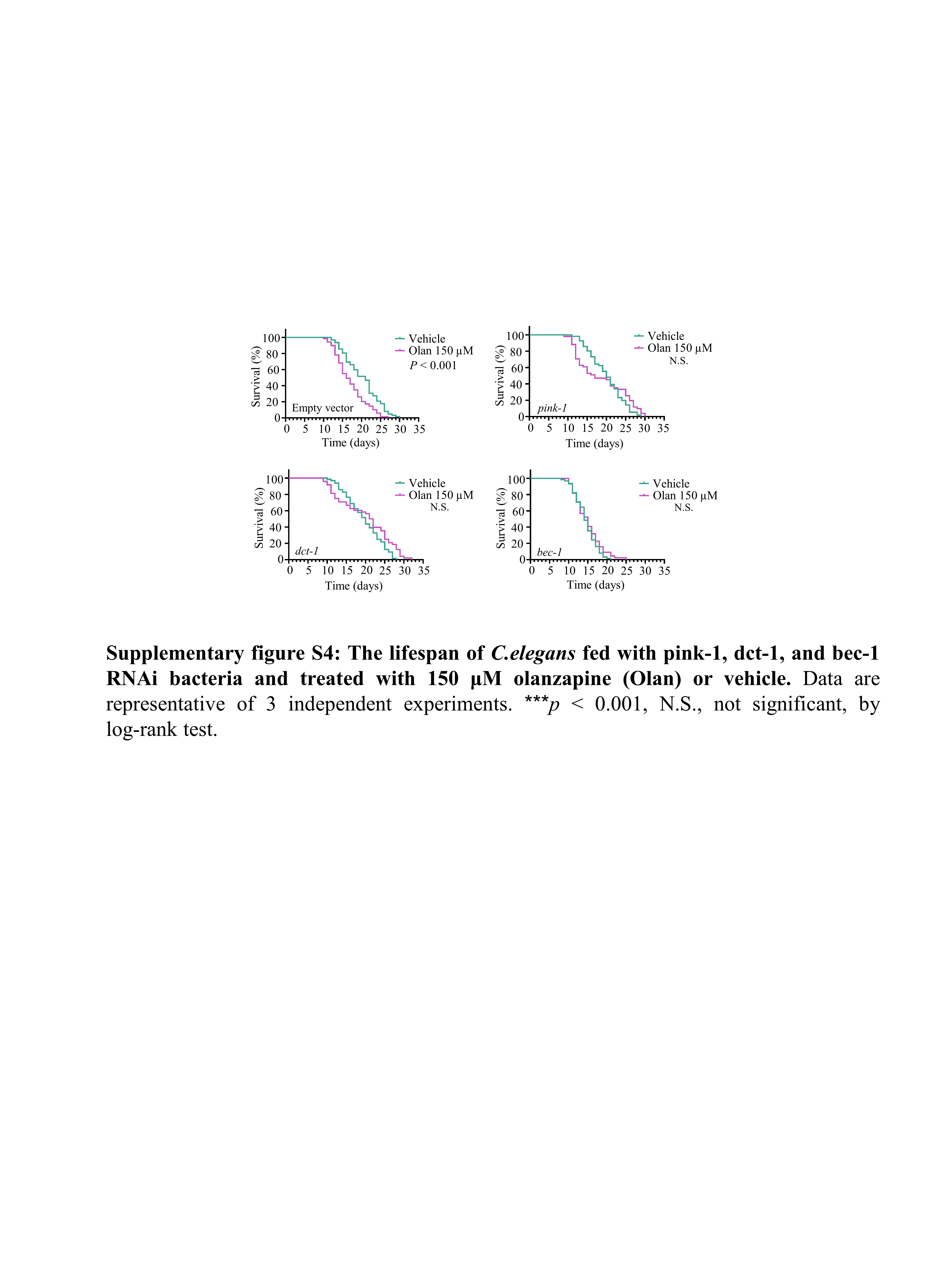

Supplement: Supplementary file 5 — Figure S4 [file ACEL-22-e14003-s007.tiff]

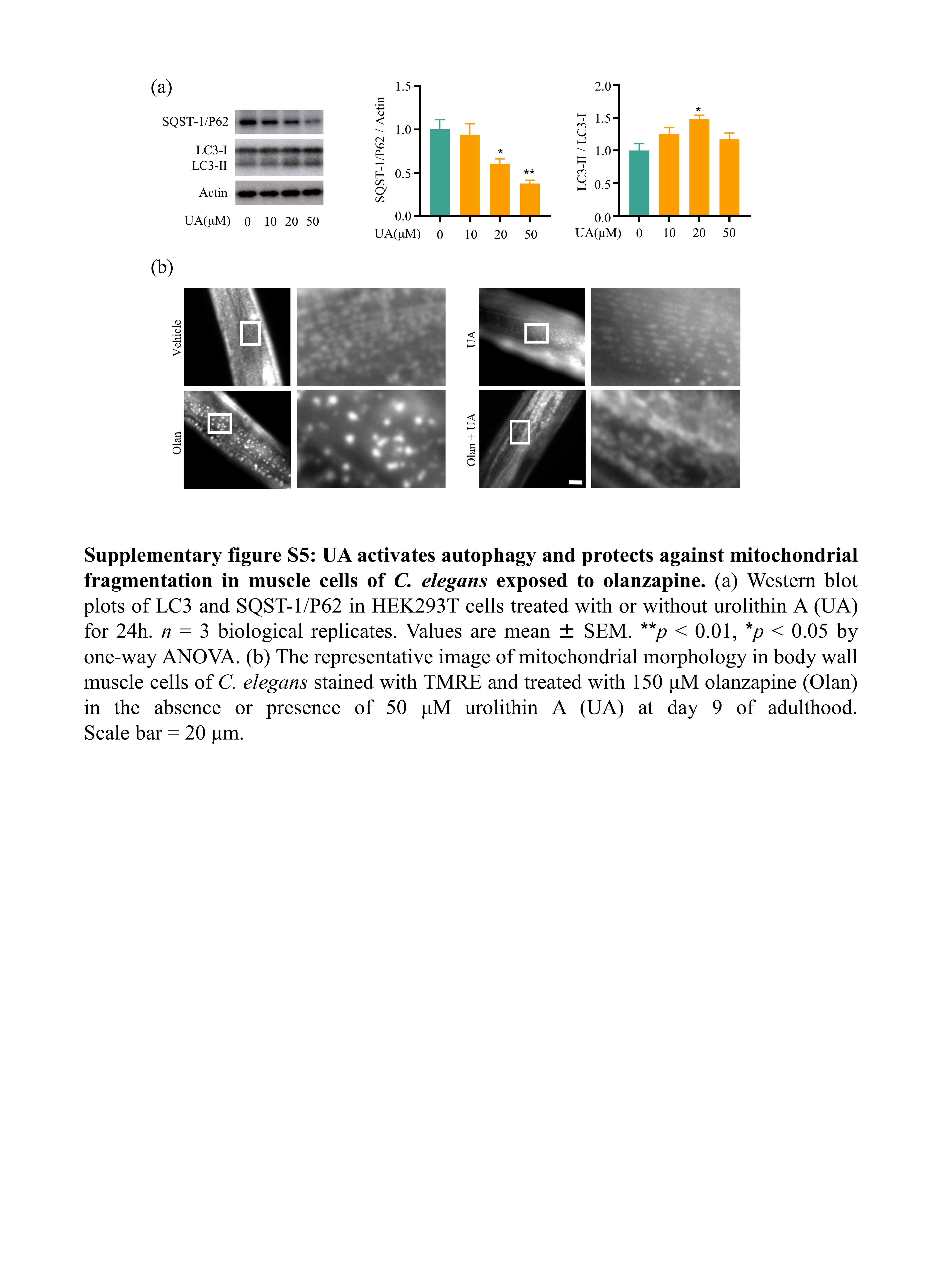

Supplement: Supplementary file 6 — Figure S5 [file ACEL-22-e14003-s004.tiff]

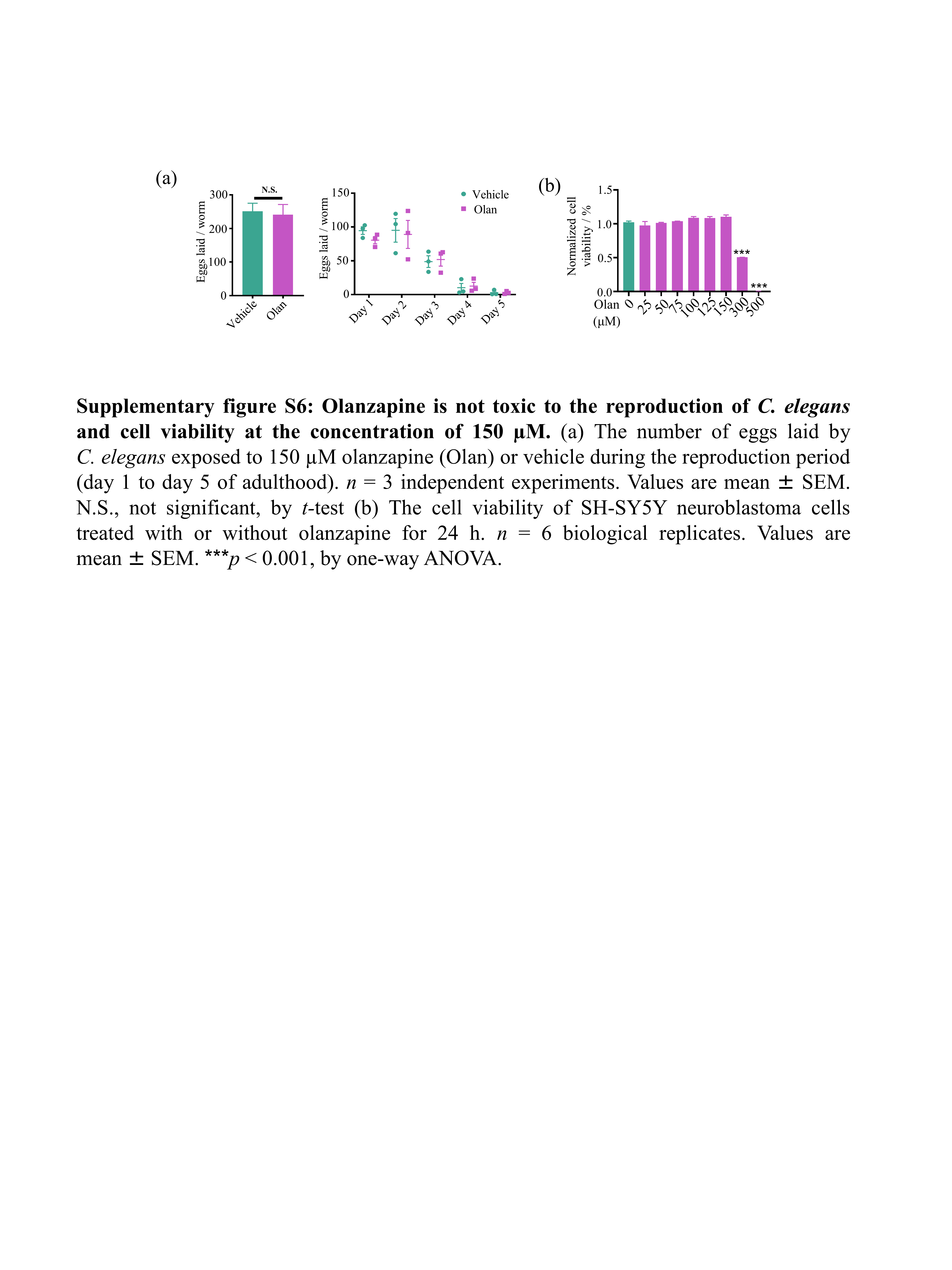

Supplement: Supplementary file 7 — Figure S6 [file ACEL-22-e14003-s001.tiff]
